# The genetics of myelodysplastic syndromes and the opportunities for tailored treatments

**DOI:** 10.3389/fonc.2022.989483

**Published:** 2022-10-20

**Authors:** Christina-Nefeli Kontandreopoulou, Konstantinos Kalopisis, Nora-Athina Viniou, Panagiotis Diamantopoulos

**Affiliations:** First Department of Internal Medicine, Laikon General Hospital, National and Kapodistrian University of Athens, Athens, Greece

**Keywords:** MDS, genetic mutations, spliceosome, epigenetics, transcription factors, cohesin complex, DNA repair, targeted therapy

## Abstract

Genomic instability, microenvironmental aberrations, and somatic mutations contribute to the phenotype of myelodysplastic syndrome and the risk for transformation to AML. Genes involved in RNA splicing, DNA methylation, histone modification, the cohesin complex, transcription, DNA damage response pathway, signal transduction and other pathways constitute recurrent mutational targets in MDS. RNA-splicing and DNA methylation mutations seem to occur early and are reported as driver mutations in over 50% of MDS patients. The improved understanding of the molecular landscape of MDS has led to better disease and risk classification, leading to novel therapeutic opportunities. Based on these findings, novel agents are currently under preclinical and clinical development and expected to improve the clinical outcome of patients with MDS in the upcoming years. This review provides a comprehensive update of the normal gene function as well as the impact of mutations in the pathogenesis, deregulation, diagnosis, and prognosis of MDS, focuses on the most recent advances of the genetic basis of myelodysplastic syndromes and their clinical relevance, and the latest targeted therapeutic approaches including investigational and approved agents for MDS.

## 1 Introduction

Myelodysplastic syndromes (MDS) comprise a heterogeneous group of malignant hematopoietic stem cell (HSC) disorders characterized by ineffective hematopoiesis, cytopenias, and an increased risk of transformation to acute myeloid leukemia (AML) (1, 2). Patient disease course, progression, and survival rates are highly variable, based on genomic features, risk stratification, symptom burden, and comorbidities. The incidence of MDS is higher in elderly patients, highlighting the increased risk of acquired mutations in hematopoietic stem cells (HSCs) and progenitor cells (HSPCs), which keep on accumulating with age and reach on average 5–10 mutations (3). The International Prognostic Scoring System (IPSS) (4) and the Revised IPSS (IPSS-R) (5) are the most widely used prognostic tools for predicting the risk of leukemic transformation and overall survival (OS). Patients with lower-risk MDS (LR-MDS) are managed largely with supportive care and agents targeting cytopenias (6). Patients with higher-risk MDS (HR-MDS) are treated with the hypomethylating agent (HMA), 5-azacytidine (5-AZA). Allogenic hematopoietic stem cell transplant (HSCT) remains the only curative treatment for younger, fit HR-MDS patients (7).

The genomic landscape of MDS revealed by next-generation sequencing indicates that mutations of various genes mainly involved in RNA splicing, epigenetic, signaling, transcription, DNA repair, and cohesin pathways serve as drivers for MDS. The rapid technological development for understanding the genetic mutations over the last decade has changed the knowledge of MDS pathogenesis and prognosis based on the discovery of higher-incidence genetic abnormalities in MDS. In this review, we give an outline of the molecular mutations involved in the pathogenesis of MDS, and their clinical implications in treatment outcomes and prognosis, sole or through interaction in co-occurrence. Moreover, we provide data on the currently available or under investigation targeted therapies for MDS. The paper is structured in a way that each molecular disorder is analyzed in terms of frequency, effect on normal protein function, clinical and prognostic impact, and a second section follows analyzing the already available or upcoming agents against individual molecular targets.

## 2 Molecular pathogenesis

The molecular pathogenesis of MDS is complex due to multiple genetic and epigenetic aberrations resulting in the dysplastic and proliferative features of MDS. These aberrant alterations are more often due to somatic mutations followed by chromosomal abnormalities and less often due to germline mutations (8, 9).

### 2.1 Cytogenetic (chromosomal) abnormalities

The hematopoietic cells of about 50% of MDS patients bear chromosomal abnormalities (10). Most of them affect genomic balance, such as deletion, monosomy or trisomy, and structural alterations of chromosomes, such as the translocations frequently seen in acute leukemia. In MDS patients with an abnormal karyotype, more than 680 different types of chromosomal abnormalities have been observed, with loss or deletion of chromosome 5 or 7 and mosaic trisomy 8 being the most common among them (11). Monosomy 7 is known to be associated with poor prognosis (11). MDS with isolated del (5q), also known as 5q- syndrome, is a unique subtype associated with a favorable outcome and characterized by macrocytic anemia with normal/high platelet counts (12). The frequency of 5q- syndrome is relatively high in the US and European countries, accounting for approximately 5% of MDS patients (13). Several pathogenic genes have been identified in the commonly deleted region of 5q31-5q33, including ribosomal protein S14 (RPS14), microRNA (miR)-145, and miR146a. RPS14 is believed to be responsible for macrocytic anemia (14), while miR-145 and miR-146a are involved in thrombocytosis and neutropenia through interleukin (IL)-6 regulation (15). Apart from 5q- syndrome, there are no other cytogenetically defined MDS subtypes.

### 2.2 Genetic mutations

As mentioned above, approximately half of MDS patients have a normal karyotype, suggesting that genetic mutations are responsible for the pathogenesis of MDS. A detailed understanding of the mutational landscape of MDS has emerged over the last years, with the advent of advanced high-throughput techniques such as multiple ligation dependent probe amplification (MLPA), high-fidelity single nucleotide polymorphism (SNP) array, next-generation sequencing (NGS), whole-genome sequencing (WGS), and whole exome sequencing (WES) (16). NGS is superior to cytogenetics in establishing clonality, especially given the high rate of a normal karyotype; thus a myeloid mutational panel can provide essential evidence of clonality, besides cytogenetics/FISH studies, useful in the differential diagnosis of cytopenia (17). Some of the recurring chromosomal abnormalities and somatic mutations observed in MDS have a distinctive gene expression profile (GEP). Nevertheless, the pathophysiology of these disorders remains not fully understood and only few *in vivo* models are available to help uncover the disease (18). Application of these technologies has revealed a large set of recurrently mutated genes which are organized in eight major categories corresponding to the implicated cellular processes: RNA splicing, DNA methylation, chromatin modification, the cohesin complex, transcription factors, the DNA damage response pathway, signal transduction and other pathways (19–21). Somatic mutations in MDS-related genes have been identified in healthy individuals with clonal hematopoiesis of indeterminate potential (CHIP) (22, 23). Similarly, patients with idiopathic cytopenias of undetermined significance, with low blood cell counts, normal karyotype, and absence of significant dysplasia, have somatic mutations in MDS-related genes in more than one-third of the time (24). Despite the inter-patient variation, some of the mutations have developed independent expression and prognostic value such as the splicing factor 3 subunit 1 (*SF3B1)* mutation with ring sideroblasts (RS) (25) in 60%-80% of RS-MDS (26) and the association of tet methylcytosine deoxygenase 2 (*TET2*) mutation with longer OS, good prognosis (27), phenotype of the disease (28), and an increased response to 5-AZA (29, 30). There are only a few genes in MDS that are mutated in high frequency, while many more are found mutated only in a small proportion of patients, with the overall pattern of most frequent mutations comprising *SF3B1, TET2, ASXL1, SRSF2, DNMT3A, RUNX1, U2AF1, ZRSR2, STAG2, TP53, EZH2, CBL, JAK2, BCOR, IDH2, NRAS*, and *NF1* genes (31–34) in order of descending frequency.

## 3 Somatic mutations

As mentioned above mutations found in MDS have been categorized into 8 subgroups depending on their function, including RNA-splicing machinery: *SF3B1, SRSF, ZRSR2, U2AF1, U2AF2, SF1, PRPF8, LUC7L2, DDX41*; DNA methylation: *TET2, DNMT3A, IDH1/2*; chromatin modification: *ASXL1, EZH2, KMT2, SUZ12, JARID2, KDM6A, PHF6, EED, EP300*; signal transduction: *FLT3, JAK2, MPL, GNAS, KIT, CALR, CSF3R, NOTCH1/2, KRAS, NRAS, CBL, NF1, PTPN11*; transcription factors: *TP53, PPM1D, RUNX1, ETV1, GATA2, ETV6, CUX1, IRF1, CEBPA, BCOR LAMB4*; cohesin complex: *STAG2, CTCF, SMC1A, RAD21, SMC3; DNA repair: ATM, BRCC3, DLRE1C, FANCA-L, BRCA2, RAD21*; others: *SETBP1, NPM1, WT1* (29,36,37,38). The molecular landscape of these mutations is shown in **Table 1**. Moreover, **Table 2** summarizes the preclinical data on targeting selective mutations, while **Supplementary Table 1** comprises the clinical trials on investigational or already approved targeted therapies in MDS.

**Table 1:** Somatic mutations in MDS.

Gene function	Gene symbol (name)	Location	Frequency (%)	Normal protein function	Mutation impact on protein function
RNA splicing	SF3B1 (Splicing factor 3b, subunit 1)	2q33.1	12-75	Core part of the U2 snRNP that recognizes the 3’ splice site at intron-exon junctions	Missense; possible dominant negative or gain of function; mitochondrial ferritin overload and formation of ring sideroblasts (RS)
	U2AF1 (U2 small nuclear RNA auxiliary factor 1)	21q22.3	7-17	U2 small nuclear RNA splicing factor that recognizes AG nucleotides in pre-mRNA slice sites	Missense; impairs erythroid differentiation and autophagy
	SRSF2 (Serine/arginine-rich splicing factor 2)	17q25.1	12-15	Pre-mRNA splicing factor that promotes exon recognition during splicing	Missense; changes RNA-binding preference of SRSF2
	PRPF8 (Pre mRNA processing factor 8)	17p13.3	1-4	Sister chromatid cohesion	Missense; defects in proof reading functions
	LUC7L2 (LUC7 Like 2, pre m RNA splicing factor)	7q34	NA	Encodes a protein that contains a C2H2-type zinc finger, coiled-coil region and arginine and may bind to RNA *via* its arginine/serine domain	NA
Epigenetic modifiers	TET2 (Tet methylcytosine deoxygenase 2)	4q24	12-23	Link between intracellular metabolism and epigenetic regulation	Nonsense; reduction of 5-hmc levels and DNA hypermethylation
	DNMT3A [DNA (cytosine-5-)-methyltransferase 3 alpha]	2p23	8-18	DNA methyltransferase	Missense (mostly); reduction of methyltransferase and subsequent hypomethylation
	ASXL1 (Additional sex combs like 1)	20q11	14-29	Chromatin binding protein; H3K27 and H3K4 methylation	Missense; recission of the C-terminus, assumed to mediate chromatin modification
	EZH2 (Enhancer of zeste homolog 2)	7q35-36	6-7	Histone-methylating protein	Missense in the SET domain; abnormal splicing leads to C-terminus recission that impairs the methyltransferase function
	IDH1 (Isocitrate dehydrogenase 1)	2q33.3	2-4	Converts isocitrate to α-ketoglutarate in the citric cycle (cytoplasm)	Missense; generates D2HG instead of αKG; inhibits the normal function of TET2, H3K9 demethylases, and cytochrome c oxidase
	IDH2 (Isocitrate dehydrogenase 2)	15q26.1	2-9	Converts isocitrate to α-ketoglutarate in the citric cycle (mitochondria)	Missense; generates D-2-hydroxyglutarate instead of α-ketoglutarate; inhibits the normal function of TET2, H3K9 demethylases, and cytochrome c oxidase
Signaling	NRAS [Neuroblastoma RAS viral (v-ras) oncogene homolog]	1p13.2	4-6	Signal transducer controlling cell growth, migration, adhesion, and differentiation	Missense; constitutive RAS activation, leading to cell proliferation
	KRAS (Kirsten RAS viral (v-ras) oncogene homolog)	12p12.1	1	Signal transducer controlling cell growth, migration, adhesion, and differentiation	Missense; constitutive RAS activation, leading to cell proliferation
	JAK2 (Janus kinase 2)	9p24	5	Tyrosine kinase that activates JAK/STAT pathway	Missense; constitutive activation of JAK/STAT pathway
	FLT3 (Fms-related tyrosine kinase 3)	13q12	2-5	Tyrosine kinase, class III receptor in progenitor cells	Missense; ligand-independent activation; increased proliferation
Transcription factors	TP53 (Tumor protein 53)	17p13.1	8-13	Regulates cell cycle, DNA repair, apoptosis, and expression of stress response genes	Missense; increased DNA damage
	RUNX1 (Runt-related transcription factor 1)	21q22.3	6-15	Member of transcription protein complex, contributing to hematopoietic self-renewal, expansion, and differentiation	Missense; reduces DNA binding and CBFβ heterodimerization
Cohesin complex	STAG2 (Stromal antigen 2)	Xq25	6-8	Regulates separation of sister chromatids during cell division	Indel; alteration of chromatin accessibility or modulation of chromatin structure
DNA repair	ATM (Ataxia telangiectasia mutated)	11q22-23	<10	DNA double strand break response; CDK-mediated phosphorylation and Cdc6 removal; apoptosis	NA
Other mutations	SETBP1 (SET binding protein 1)	18q21.1	2-5	Cell division	Missense; cell proliferation *via* inhibition of PP2A

C2H2-type zinc finger, cys2/his2-type zinc finger; CBFβ, core-binding factor subunit beta; CDc6, cell division cycle 6; CDK, cyclin-dependent kinase; H3K4, histone methyltransferase-lysine 4; H3K27, histone methyltransferase-lysine 27; JAK/STAT, Janus kinase and signal transducer and activator of transcription; PP2A, protein phosphatase 2; snRNP, small nuclear ribonucleic particles; NA, Not applicable.

**Table 2:** Preclinical data on agents targeting mutations commonly found in patients with myelodysplastic syndrome.

Category	Target	Agents	Intervention	Reference
RNA splicing	*SF3B1*	Pan splicing modulators (FR901463, FR901464, and its chemical product spliceostatin A, FR901465)	Inhibit *in vitro* splicing and promote pre-mRNA accumulation by SF3b binding, preventing the recruitment of U2 snRNP to the branch point and destabilize it.	(35–37),
		Sudemycin D6 (FR901464 analog)	*In vitro* treatment of human CD34+ cells results in expression differentiation and splicing dysregulation (sudemycin modifies rather than inhibits splicing)	(38)
		H3B-8800	Apoptosis induction in *SF3B1* mutant cells	(35, 38, 39)
	*U2AF1*	Sudemycin D6 (FR901464 analog)	Overexpression of *U2AF1* ^S34F^ leads to increased sensitivity to sudemycin D6	(38)
	SRSF2	E7101 (derivative of pladienolide B)	Induces apoptosis in *SRSF2* mutant cells	(35)
	*RBM39*	E7070, E7820	Induce proteasomal degradation of the splicing factor RBM39	(40–42)
	*PRMT5*	GSK591	PRMT5 and type I PRMT inhibition preferentially affects SF mutants (SRSF2-P95H)	(43)
Epigenetic mutations	*DOT1L*	SYC-52221	Decrease tumor cell proliferation and induce cell apoptosis, cycle arrest, and terminal differentiation in DNMT3A mutant murine cell lines in a dose- and time-dependent manner	(44)
		EPZ004777		
		EPZ5676	Efficacy in a nude mouse xenograft model of AML with mutant DNMT3A.	(44)
	*TET2*	HMAs	Patients harboring *TET2* mutations but lacking ASXL1 mutations, show high response rates to HMAs	(45)
	IDH1-R132	AGI-5198	Blocks the production of D2HG by mutant cells resulting in the restoration of differentiation and proliferation in leukemic cells	(46)
	IDH1	HMS-101	Induces cell death within bone marrow cells expressing mutant IDH1 decreasing the mutant population.	
	*IDH1*	Vitamin C	Decreases proliferation of leukemic cells with mutant *IDH1* and increases expression of genes associated with differentiation	(47)
	*IDH1*	Ivosidenib (AG-120)	Target mutant IDH1 and IDH2 by blocking D2HG, leading to reduced blast count and increase in mature myeloid cells	(46)
	*IDH2*	Enasidenib (AG-221)		
	*IDH2*	AGI-6780	Promotes cellular differentiation with a decrease in myeloblasts and increase in mature monocytes and granulocytes	(48)
	*TET2*	Vitamin C	Induces clinical remission in AML and is probably associated with driver mutations in the *IDH1/2-TET2-WT1* pathway	(47)
	*EZH2*	EI1	Inhibits cell growth and induces apoptosis and differentiation in lymphoma cells by reduction of H3K27me3 levels	(49)
	HDAC	Vorinostat	Reverses histone acetylation around the promoter regions of multiple TGFβ pathway genes and induces their transcriptional derepression with simultaneous growth-inhibition effect in ASXL1 mutants	(50)
Transcription factors	P53	APR-246	Binds to cysteine residues of the core domain of mutated p53, resulting in restoration of its active formation.Induces oxidative stress by depletion of glutathione and inhibition of thioredoxin reductase, thus increasing accumulation of reactive oxygen species and further promoting tumor cell death	(51, 52)
	TP53	APR-246 alone or in combination with 5-AZA	Induce apoptosis in TP53 mutants through p53 reactivation in MDS and AML cell lines	(53)
Signal transduction	TGF-βR	LY-2157299	Anti-tumor activity including inhibition of tumor cell migration and mesenchymal phenotype, reversal of TGF-β-mediated immune-suppression, and tumor growth delay	(54)
Cohesin complex	STAG2	PARPi	Increased levels of DNA damage and sensitivity of cohesin-mutant cells	(55)
DNA repair	ATM	ATMi	Synthetic lethal DNA damage response defects may make the affected tumors more susceptible to ATMi therapy than normal tissues	(56)
Other mutations	*SETBP1*	Vorinostat	Reverses histone acetylation at these promoter regions and induces transcriptional derepression of the TGF-β pathway genes with a simultaneous growth-inhibition effect in SETBP1 mutants	(50)

5-AZA, 5-azacytidine; AML, acute myeloid leukemia; ASXL1, Additional sex combs like 1; ATM, ataxia-telangiesctasia mutated; ATMi ataxia-telangiesctasia mutated inhibitors; D2HG, 2-hydroxyglutarate; DNMT3A, DNA (cytosine-5-)- methyltransferase 3 alpha; DOTL1, DOT1-like, histone methyltransferase; EZH2, Enhancer of zeste homolog 2; H3K27me3, histone methyltransferase responsible for tri-methylation of lysine 27; HDAC, histone deacetylase; HMAs, hypomethylating agents; IDH1/2, Isocitrate dehydrogenase 1/2; MDS, myelodysplastic syndrome; P53, protein 53; PARPi, (ADP-ribose) polymerase inhibitors; PRMT5, protein arginine methyltransferase 5; RBM39, RNA binding motif protein 39; SETBP1, SET binding protein 1 gene; SF3B1, splicing factor 3b, subunit 1; snRNP, small nuclear ribonucleic particles; SRSF2, Serine/arginine rich splicing factor 2; STAG2, stromal antigen 2; TET2, tet methylcytosine deoxygenase 2; TGFβR, transforming growth factor beta receptor; TP53, tumor protein 53; U2AF1, U2small nuclear RNA auxillary factor 1; WT1, Wilms Tumor 1.

### 3.1 RNA-splicing machinery

Spliceosome is a protein complex, composed of five small nuclear RNAs (snRNAs) and approximately 150 proteins, required for the splicing reaction (57). The complex is responsible for the removal of non-coding sequences (introns) from precursor mRNA (pre-mRNA) to form mature mRNA by ligating the coding sequences (exons). There are two types of pre-mRNA introns: U2-dependent (major spliceosome), which accounts for almost all the human introns, and U12-dependent (minor spliceosome), which accounts for less than a thousand introns (58). The U2-auxiliary factor (U2AF) protein consists of a U2AF35 (U2AF1) subunit and a U2AF65 (U2AF2) subunit. U2AF binds to SF3B1 through a splicing factor (SF1) to form a heterodimer complex. The U2AF1/U2AF2 heterodimer has a high affinity for Zinc finger RNA binding motif and serine/arginine rich 2 (ZRSR2) and serine/arginine rich splicing factor (SRSF2) (59). Any alterations to the spliceosome complex can result in a change in splicing specificity, leading to alternative splicing outcomes.

#### 3.1.1 RNA-splicing machinery mutations

Splicing factor mutations are detected in 45%-85% of MDS, occur in a mutually exclusive manner, and are predominantly heterozygous. This indicates that homozygous splicing factor mutations are likely to be lethal to the cell (60, 61), missense, and located in restricted regions (62). *SRSF2* is found mutated in a frequency of about 12%. Another 20% of MDS patients carry mutations in genes, such as *U2AF1*, *U2AF2*, *SF3B1*, and *ZRSF2*. They co-occur with epigenetic mutations, indicating cooperation of these mutations, and resulting in a specific phenotype. Mutations in *SRSF2* are frequently concomitantly present with runt-related transcription factor 1 (*RUNX1)*, isocitrate dehydrogenase 2 (*IDH2)*, additional sex combs like 1 (*ASXL1), TET2* and *(STAG2)* mutations, where co-mutation of *TET2* and *SRSF2* has been found predictive of leukemic transformation in MDS and CMML (63). Co-occurrence of mutations in *ZRSR2* and *TET2*, as well as *SF3B1* and DNA (cytosine-5-)- methyltransferase 3 alpha (*DNMT3A*), or *U2AF1* and *ASXL1* or *TET2* have been noted (27). Two recent studies have used RNA sequencing to show that the common splicing factor mutations result in different alterations in splicing and affect different genes (64, 65). Enhancer of zeste homolog 2 (*EZH2)* in *SRSF2* and *U2AF1* mutant MDS cases has been found aberrantly spliced, thus representing a common target. *EZH2* among other aberrantly spliced genes identified in both studies, is known to be involved in MDS pathogenesis, because of being recurrently mutated or involved in translocations. It has been also shown that *SF3B1* mutations can be second hit mutations following epigenetic mutations and that they cooperate with *EZH2* mutations to impair mitochondria and induce apoptosis (45). The presence of mutational hot spots and frameshift derangement could be correlated with gain or change of function of splicing factor genes leading to MDS. Concurrent spliceosome gene mutations, such as *SRSF2* and *SF3B1*, are associated with HR-MDS, higher bone marrow blast percentage, and deregulation of RNA splicing and DNA methylation pathways (66).

The high mean variant allele frequency (VAF) of *SF3B1* found in studies shows that the *SF3B1* mutation is present in dominant MDS clones, suggesting that the mutation itself originates from cells actively proliferating in the MDS clones (66), while it has been also proposed that the consecutive acquirement of genetic lesions with *SF3B1* in one of the mutated major clones at the early stages of MDS is implicated in AML transformation (67). Despite the mutual exclusivity of mutations in spliceosome members, mutations in *SF3B1* appear to be markedly high in MDS-RS with an incidence of 60%-80% in these patients, but only 10–20% of unselected MDS. It is currently known that *SF3B1*-mutated MDS-RS patients, may have better outcomes compared with their wild-type partners (20, 26). *SF3B1^+/-^* mice accumulate RS in the bone marrow, which are rarely found in animal models of MDS, suggesting that *SF3B1* haploinsufficiency causes biological features resembling low risk MDS-RS indicating that this mouse model could be a target for preclinical therapeutic studies (68). Clonal analysis has shown that *SF3B1* mutations occur in rare lympho-myeloid HSCs (Lin− CD34+ CD38− CD90+ CD45RA−) providing a distinct clonal advantage to MDS-RS HSCs (68). However, it is widely recognized that any phenotypically defined HSC population also contains a substantial fraction of non-HSCs, and since the lymphoid lineages have not been found to be involved in the *SF3B1*-mutated clones, definitive evidence for recurrent *SF3B1* mutations targeting true lymphomyeloid HSCs in MDS-RS is lacking (69, 70). Several studies have identified, using RNA-sequencing, deregulated gene isoforms and aberrantly spliced target genes in *SF3B1* mutant MDS (71–74). The most studied gene which has been shown to be aberrantly spliced in SF3B1 mutant MDS-RS, is the mitochondrial iron exporter ATP binding cassette subfamily B member 7 (ABCB7), since a strong association between *SF3B1* mutations and *ABCB7* down-regulation has been found (69). Several studies have investigated genes involved in iron homeostasis and hemoglobin biosynthesis in *SF3B1* mutant MDS and identified cryptic splicing events of them (72, 75, 76). As an example, *SF3B1* mutations are often associated with alternative splicing of solute carrier family 25 member 37 (*SLC25A37*), a splice variant upregulated in MDS-RS patients with thrombocytosis, which acts as a significant importer of iron in the mitochondria (77) with a vivid role in mitochondrial iron delivery in erythroid cells (78). Somatic mutations of the pre-mRNA processing factor 8 (*PRPF8)* splicing gene that are also responsible for abnormal splicing involved in the iron metabolism have been also reported (79–81). Luc7-like 2 gene (*LUC7L2)* is located on chromosome 7q34 and has been found aberrantly spliced in MDS cases harboring *SRSF2* small deletions. These mutations can be heterozygous, homozygous, and hemizygous and although they are not well characterized, it seems that they have been associated with short survival in patients with -7/del17q (82).The fact that *SF3B1* mutations delineate a distinct type of MDS imply that the effects of RNA splicing factors mutations on mis-spliced target genes may open a whole new field for research.

#### 3.1.2 Splicing as a therapeutic target

Observations of the survival dependence of splicing factor mutants on the wildtype splicing function (61, 62) have motivated the pharmacologic interest for targeting splicing. Several compounds are known to target specific steps in pre-mRNA splicing and catalysis. Pan-splicing modulators such as bacterially derived products and analogs have been shown to bind the SF3B complex and disrupt spliceosome balance (35, 83), while others such as FR901463, FR901464, and FR901465, have been shown to bind and inhibit *SF3B1* from binding the pre-mRNA, thus preventing the recruitment of U2 snRNP to the branch point and destabilize it. The first that was identified was FR901464 and its chemical product, spliceostatin A (36). Meayamycins, sudemycins and thailanstatins (37, 84, 85)are analogous compounds that belong to the FR901464 family of splicing inhibitors. Specifically, *in vitro* treatment of human CD34+ cells with sudemycin D6 resulted in 1.030 differentially expressed genes and 18.833 dysregulated splicing events (86) leading to the conclusion that sudemycin mostly modify rather than inhibit splicing. Moreover, it has been observed that overexpression of *U2AF1* ^S34F^ lead to an increased sensitivity to sudemycin D6 and that this may be due to aberrant splicing caused by the drug. Other splicing modulators like FR901464 are the pladienolide inhibitors (87). E7101, a synthetic derivative of pladienolide B and more recently H3B-8800, a selective and orally bioavailable modulator of wild type and mutant SF3B seem to induce apoptosis in *SRSF2* (38) and *SF3B1* (38) mutants respectively. In an ongoing. phase I study (NCT02841540) of patients with myeloid cancers, different doses of H3B-8800 were compared in 84 patients with most of them harboring spliceosome mutations. The results have so far demonstrated dose-dependent target engagement, predictable pharmacokinetics profile, and safety even with prolonged dosing (35). Splicing modulators with different mechanism of action have also been identified. Idole derivatives (benzopyridoindoles) can modulate splicing (39), and small molecule kinase inhibitors (e.g., TG003 and SRPIN340) can modulate post-translational modification of serine-arginine rich proteins by blocking their interactions that are crucial for normal splicing (88).

In addition to these compounds that mostly inhibit the enzymatic activity of splicing, molecules that modify the abundance of splicing proteins are also gaining their place in the therapeutic approach of targeting RNA splicing. Recently, a subclass of sulfonamides including E7070, E7820, indisulam, tasisulam, and chloroquinoxaline sulfonamide (40, 41, 89) were shown to induce proteasomal degradation of the splicing factor RNA binding motif protein 39 (RBM39). RBM39 is a serine-arginine rich RNA binding protein, that shares great sequence with U2AF2 and is involved in the binding of the spliceosome to the 3’ exon, which is crucial for appropriate splicing (42, 90). Taking into consideration that spliceosome mutant leukemias seem to be sensitive to inhibition of protein arginine methyltransferases (PRMTs), a study identified that inhibition of arginine demethylation mediated by PRMT5 leads to preferential death of splicing factors mutant cells (91). The use of CRISR/Cas9 revealed that inhibition of the RBM39 protein leads to mis-splicing of Homeobox A Cluster (*HOXA9*) target genes, which constitute a network essential for AML survival (43).

Oligonucleotide-directed approaches such as RNA interference (RNAi) (92) and antisense oligonucleotides (ASOs) are also under development. A phase I trial (NCT01159028) investigated the safety and toxicity of BP1001, a liposomal growth factor receptor bound protein-2 antisense oligodeoxynucleotide (L-Grb2 AS) for patients with Philadelphia chromosome positive CML, AML, ALL, and MDS. BP1001 was well tolerated, with more than half of evaluable patients experiencing ≥50% reduction in peripheral or bone marrow blasts (93). A major challenge of using ASOs therapeutically is the improvement of their delivery systems. Recently, studies have revealed a strong correlation between splicing factor mutations and elevated levels of R-loops, DNA-RNA intermediates, and activation of the ataxia telangiectasia and Rad3-related protein (ATR) pathway (94, 95). DNA damage and cell death seem to be caused by pharmacological inhibition of ATR in CD34+ MDS cells. This can be enhanced by combination treatment with the low dose splicing modulatory compound Pladienolide B (95, 96).

### 3.2 Epigenetic mutations

Epigenetic modifications, such as DNA methylation and histone modification, result in inheritable changes in gene expression without changing the DNA sequence. Genes involved in DNA methylation and chromatin modification make up a second common class of mutations in MDS.

#### 3.2.1 DNA methylation

Deregulation of CpG island methylation within gene promoters is a major epigenetic transcriptional control mechanism in human solid tissue and hematologic tumorigenesis (96, 97). Epigenetic changes in *DNMT3A, IDH1/2, and TET2* are frequently reported in MDS causing hypermethylation, adhesion, and disease specific changes in HSCs.

Recurrent missense, nonsense, and frameshift mutations have been identified in DNMT3A, a methyltransferase enzyme that catalyzes the transfer of methyl groups to cytosines in CpG islands of DNA (98) DNMT3A is recurrently mutated in *de novo* AML and at low frequency in MDS with dominance at older age. These mutations of the methyltransferase domain are heterozygous and mostly missense or nonsense. The presence of *DNMT3A* mutations in nearly all bone marrow cells of MDS patients independent of blast count, has led to the hypothesis that this is an early genetic event in the disease process and may result in a clonal advantage of the mutated cells (99). It has been demonstrated that *DNMT3A* silencing leads to progressive damage of hematopoietic stem cell differentiation and thus, plays a crucial role in silencing of regulatory genes (100). The most commonly identified mutation is the missense R882 methyltransferase domain mutation, which occurs in almost half of *DNMT3A* mutated cases. Some reports have shown that the 5-year survival of AML patients with *DNMT3A* ^R882^ mutation is significantly shorter than that of AML patients without such mutations (101–103). Moreover murine models harboring *DNMTA3A* ^R878H^ did not develop leukemia and were instead characterized by an increased percentage of circulating c-Kit-positive progenitor cells, consistent with HSPC expansion (99).

TET2, is an enzyme that hydroxylates methylated cytosines to initiate the process of DNA demethylation, by converting the methyl group at the 5-position of cytosine DNA 5-methylcytocine (5mC) to 5-hydroxy-methylcytosine (5hmC) (104). Somatic *TET2* mutations in MDS are associated with advanced age, clonal hematopoiesis, and normal karyotype, suggesting that *TET2* mutation is an aging-associated factor of hematopoietic cells (100). Abnormalities in *TET2* with disabled *TET* function are observed in 15–27% of MDS, and include deletions, loss of heterozygosity, and missense, nonsense, and frameshift mutations leading to impaired DNA demethylation. *TET2* mutations have most consistently been associated with favorable responses to HMAs (105, 106). This seems to be limited to patients in whom *TET2* is an early, clonal mutation as opposed to a late subclonal event. On the contrary, *TET2* mutations can be early events in some patients with MDS and secondary AML associated with shorter OS after HSC transplantation (107). Biochemical studies (107, 108) have demonstrated reduced levels of 5hmC in the genomic DNA of bone marrow cells from patients with *TET*-mutated malignancies, implying a catalytic activity of mutant *TET2*. Interestingly, patients with low 5hmC levels display a similar clinical phenotype independent of *TET2* mutation status, suggesting that 5hmC levels rather than *TET2* mutation status, may mediate the observed clinical phenotype.

TET2 activity is also affected by mutations in isocitrate dehydrogenase 1 (*IDH1)* and *IDH2*. IDH1 and IDH2 belong to the IDH family that convert isocitrate to α-ketoglutarate (aKG) *via* oxidative decarboxylation. IDH2 is located in the mitochondria, whereas IDH1 in the cytosol and peroxisome. Heterozygous mutations alter the isocitrate dehydrogenase (IDH) enzymatic activity, resulting in the generation of 2-hydroxyglutarate (D2HG), an oncometabolite that inhibits the activity of several targets, including *TET2*. It is widely accepted that D2HG inhibits aKG-dependent dioxygenases and contributes to epigenetic alterations driving leukemogenesis (109). The connection between the two mutations became apparent when the mutant IDH enzymes were shown to catalyze the conversion of αKG to D2HG, consuming NADPH (110). D2HG is normally present at very low levels in non-mutated cells but accumulates at 100-fold higher levels in IDH-mutated AML cells (111). D2HG is structurally similar to αKG and a competitive inhibitor of αKG dependent dioxygenases including TET2 (112). The indirect interaction between *IDH* and *TET2* mutants results in increased global expression of 5mC and impaired DNA damage repair mechanisms promoting myeloid malignancies. Mutations in IDH proteins are found in 15–20% of AML cases and less commonly in MDS patients occurring in 3–5% of cases (113). They are missense alterations affecting arginine-132 (R132) in IDH1 and either the arginine residue (R172) or the arginine-140 (R140) residue in the IDH2 protein (114). *IDH1/2* mutations are generally heterozygous, suggesting a gain of function by the enzyme as a potential cancer mechanism (115). Expression of these mutations in mouse models initiate hematologic malignancy characterized by an increase in early hematopoietic progenitors, splenomegaly, anemia, hypermethylated histones, and altered DNA methylation patterns similar to those found in patients with AML with IDH1/2 mutations. It has been also shown that IDH1 mutant mice develop myeloid dysplasia *via* disturbance of heme biosynthesis and erythropoiesis, thus highlighting the essential role of IDH1 in normal erythropoiesis (116). Overexpression of mutant IDH enzymes can induce histone and DNA hypermethylation, as well as block cellular differentiation, which can be reversed by small molecule inhibition of the mutant enzymes (117–119). IDH mutations cooperate with other mutations to drive oncogenesis (120). They co-occur with nucleophosmin (*NPM1)* mutations (121), leading to better prognosis, but are almost always mutually exclusive with *TET2* mutations.

#### 3.2.2 Chromatin modifiers

Epigenetic regulation of posttranslational modifications of histone proteins at specific residues, including acetylation, methylation, and ubiquitination is catalyzed by a group of histones modifying enzymes with distinct specificity, some of which have been observed with mutations in MDS.

DOT1-like, histone methyltransferase (DOT1L), a histone lysine methyltransferase without a SET domain and its homologs are involved in numerous processes, including transcriptional regulation, cell cycle progression, and DNA damage repair, and are implicated in several cancers (122). EZH2 is the main catalytic member of the polycomb repressive complex 2 (PRC2) that also includes loss-of-function mutations in embryonic ectoderm development (*EED)*, SUZ12 polycomb repressive complex 2 subunit (*SUZ12)* and *EZH1* with a frequency less than 5%. *EZH2* encodes a histone methyltransferase that is responsible for mono-, di, and tri-methylation of lysine 27 of histone 3 (H3K27me1, H3K27me2, HEK27m3). Point mutations of these methyltransferases offer high catalytic activity to the H3K27me3 in malignant cells. *EZH*2 also interacts with *DNMT3A* as mentioned above, affecting DNA methylation. Gene somatic mutations are associated with uniparental disomy of long arm of chromosome 7, del7q, and *EZH2* reduced expression in CD34+ cells (123). When occurring in 7/7q-chromosome abnormalities, loss of function mutations induce overexpression of the HOX genes in MDS (124). Despite that *EZH*2 is commonly overexpressed in high risk MDS and AML, the observation that loss of function mutations affect patients with MDS/MPN, myelofibrosis, and various MDS entities highlights the fact that *EZH2* can function as tumor suppressor and oncogene depending on the cellular context (125). Overall *EZH2* mutations are associated with poor outcomes (126), but in MDS they are not associated with progression to AML (127). Moreover, *EZH2* mutations co-occur with *RUNX1* mutations in MDS and MDS/MPN patients (24, 27). *EZH2* loss in HSCs significantly promotes RUNX1S291fs-induced MDS (128). In the MDS bone marrow, normal hematopoietic cell development is impaired *via* the activation of the interleukin-6 pathway amongst others, which has been shown to impair normal HSCs *in vivo*, hence promoting the survival and proliferation of MDS clones. Moreover, combined inactivation of *EZH2* and *RUNX1* in progenitor cells has been found to enhance proliferation of early thymic progenitors in mice (129). *EZH2* mutations also frequently co-occur with *TET2* mutations (27, 28, 130). Mice models have revealed that *EZH2* or *TET2* deficiency alone is sufficient to induce an MDS/MPN phenotype with lethality, while depletion of both *EZH2* and *TET2* synergistically induces myelodysplasia and strengthens the progression of both MDS and MDS/MPN (114).

The *ASXL1* gene also plays a crucial role in recruiting polycomb repressive complex 2 (PRC2) to specific loci and normally encodes a chromatin-binding protein for the epigenetic regulation in hematopoietic cells. It belongs to a 3-member family (ASXL1/2/3) which is thought to mediate the balance between polycomb and trithorax functions and is involved in the maintenance of activation and silencing of *HOX* genes through chromatin remodeling (131). *ASXL1* knockout mice present impaired hematopoiesis and development of MDS-like disease through decreased H3K4me3 and H3K27me3 in specific loci such as HOXA genes due to loss of interactions with the PRC2 complex. These findings indicate that wild-type ASXL1 is a major tumor suppressor in hematopoiesis and suggest loss-of-function features of *ASXL1* mutations (132). ASXL1 wild-type protein can also support H3K4 methylation through O-linked N-acetylglucosamine transferase (OGT) (44), H2AK119 ubiquitination through PRC1, and H3K27 methylation in specific loci. Mutations in *ASXL1* were originally identified in MDS with del20q11 (133). They involve somatic nonsense and out-of-frame insertion/deletion mutations that result in loss of function. *ASXL1* is present in 15-24% of MDS patients as well as in clonal hematopoiesis of indeterminate potential (CHIP). They are three to five times more frequent in AML patients older than 60 years of age, are mutually exclusive with mutations in *NPM1* in this population, and rarely found in combination with FMS related receptor tyrosine kinase 3-internal tandem duplication (FLT3-ITD (134). *ASXL1* mutations are found to be associated with unfavorable prognosis and reduced survival among MDS patients transforming to AML independently of other clinical features, including age, cytogenetics, and cytopenias (135).

#### 3.2.3 Epigenetic targeting

The HMAs which act as DNMT inhibitors, and the histone deacetylase (HDAC) inhibitors (vorinostat, romidepsin, etinostat) are the first epigenetic inhibitors that have gained FDA approval. Currently, HMAs constitute the treatment of choice for high-risk MDS patients. The incorporation of the azanucleotides, 5-azadeoxycytidine (decitabine), or 5-AZA, which is metabolized into decitabine (DEC) into the cell, essentially brings about a demethylation state which antagonizes the hypermethylation effected by the aberrant epigenome of MDS patients. HMAs has been shown to improve the overall survival of higher-risk MDS by delaying the transformation to AML (136). Hypomethylation is also effective in inducing hematopoietic recovery in ˂50% of patients with lower-risk MDS, although it does not improve survival (137, 138). The response rate of HMA in higher-risk MDS is 40-50%, and it normally takes 3-6 cycles to obtain a substantial hematological response (139). Factors predicting response to HMA are reported to be mutations of *TET2* and tumor protein 53 (*TP53)* in high-risk groups, *DNMT3A* mutations, DNA hypomethylation, the absence of an *ASXL1* mutation, and pharmacologic factors limiting efficacy of HMAs (high expression of cytidine deaminase (139) or low expression of deoxycytidine kinase which is a rate limiting step for DNA incorporation (140)). High (ADP-ribose) polymerase 1 (PARP1) mRNA levels have been correlated with better response (141) and higher survival rates after 5-AZA (142). In a recent study, it has been shown that the combined expression of small noncoding RNAs (sncRNAs) in MDS patients harboring *SF3B1* and *DNMT3A* mutations can predict response to 5-AZA as well as define survival (143). Moreover, in a study of 98 MDS patients treated with 5-AZA, high methylation status of RNR subunit 1 (*RRM1*) was observed in responders, thus making RNR a possible prognostic factor for response to 5-AZA and for predicting success of epigenetic treatment (144).

Although inhibitors targeting *TET2* mutations have not yet been developed, patients harboring *TET2* mutations but lacking ASXL1 mutations showed high response rates to HMAs, indicating that they can be used as a predictor for treatment response in MDS patients (107). A recent study investigated the use of ascorbate (Vitamin C) treatment for *TET2* mutant AML patients and has shown its use in enabling active demethylation of DNA (145). The use of hypomethylating agents against *IDH* mutations has been evaluated by treating with DEC and 5-AZA as single therapies or in combination with vorinostat or valproic acid (146). The results showed no association between *IDH* mutation status and treatment response. Ex vivo treatment of human AML cells with AGI-6780 has shown an outbreak of cellular differentiation with a decrease in myeloblasts and increase in mature monocytes and granulocytes (147).

Guadecitabine (SGI-110) is a next-generation HMA with a prolonged half-life. It is designed as a DEC analogue, which makes it resistant to degradation by cytidine deaminase. It is currently under clinical trials for MDS and AML (47, 48). It has been shown to be effective for higher-risk MDS, CMML, and low-blast count AML after 5-AZA failure in phase 2 trials (148). Moreover, CC-486, an oral 5-AZA compound has shown promising results regarding the toxicity and effectiveness in MDS patients and has been recently approved by FDA, as maintenance therapy in AML patients in remission after intensive chemotherapy (149, 150). Another recently approved FDA drug, ASTX727 (INQOVI^®^), is a combination of oral DEC and cedazuridine (151), a fluorinated tetrahydrouridine derivative specifically designed to inhibit cytidine deaminase and facilitate oral administration of hypomethylating agents (152). In a phase 1b study, it was shown that the combination of 5-AZA with pevonedistat, which is considered to suppress the proliferation of leukemic cells, is effective for AML (153). A potential survival benefit from rigosertib for a small group of high-risk MDS patients with HMAs failure within the first nine months was suggested, despite that the results from a randomized phase 3 clinical trial were not encouraging (154).

Ivosidenib (AG-120) and enasidenib (AG-221) target mutant IDH1 and IDH2 respectively, by blocking 2-hydroxyglutarate (2-HG) leading to a reduced blast count and an increase in mature myeloid cells. Overexpression of D2HG can also be targeted with AGI-5198 (selective inhibitor targeting IDH1-R132) by blocking the production of D2HG by mutant cells resulting in the restoration of differentiation and proliferation in leukemic cells (155). Another inhibitor, HMS-101, targeting IDH1 has been developed and showed to induce cell death within bone marrow cells expressing mutant *IDH1* decreasing the mutant population (156). High levels of methylation of histone 3 at lysine 79 (H3K79me) are detected in AML patients bearing *MLL* rearrangements (MLL-r).

A recent study showed that SYC-52221 and EPZ004777 inhibitors that target DOT1L, decrease tumor cell proliferation and induce cell apoptosis, cycle arrest, and terminal differentiation in *DNMT3A* mutant cell lines in a dose- and time-dependent manner (46). Furthermore, the DOT1L inhibitor EPZ5676 showed promising efficacy in a nude mouse xenograft model of AML with mutant *DNMT3A* (44).

EZH2 has been considered a novel target for identification of small candidate compounds that can inhibit EZH2 activity. EI1, an EZH2 inhibitor in lymphoma, is currently under development and it seems to (157) inhibit cell growth and induce apoptosis and differentiation in lymphoma cells by reduction of H3K27me3 levels (158). Targeting genes which are derepressed because of *EZH2* or *PRC2* loss-of-function, such as HOXA9 may provide a successful therapeutic strategy. Inhibition of HOXA9 is achievable either on an epigenetic level through inhibition of expression or directly through inhibition of the HOXA9 transcription factor function, i.e., through targeting protein/protein or protein/DNA interactions. Several HOXA9 inhibitors such as HXR9 are already under clinical evaluation for patients with AML, highlighting the role of HOXA9 in leukemogenesis (159). There may also be therapeutic potential for exploring synthetic lethality, where there is mutational loss of EZH2. In mice, EZH1 has been shown to compensate for loss of EZH2 by repositioning of EZH1 to EZH2 targets (49, 158). Overall, EZH2 aberrations and PRC2 deregulation seem to be extremely prevalent in myeloid leukemia, hence, targeting EZH2 dysfunction directly or indirectly seems to be a promising approach for many patients.

Efficacy of lenalidomide on del5q in MDS is another example (160). Lenalidomide has been approved for low and intermediate risk MDS patients with 5q- (161, 162). In patients with 5q-, treatment with lenalidomide results in cytogenetic complete remissions and lower transfusion requirements (162). Inhibition of cell cycle and apoptosis, emerging from this molecule, leads to an anti-proliferative effect on leukemia blasts and a modulation effect on the bone marrow microenvironment immune cells in MDS. Recent studies have also shown that lenalidomide acts through a karyotype-dependent manner modulating the expression of genes already haploinsufficient (53, 161, 163). Moreover, patients who do not respond to lenalidomide are at a greater risk for clonal evolution to leukemia compared to responding patients (164). Surprisingly, lenalidomide was shown to have clinical activity in 20–30% of non-5q31 deleted MDS patients (165). Nevertheless, even within the subgroup of 5q- patients with a generally favorable prognosis, TP53-mutated patients tend to have decreased response rates and increased risk of evolution to leukemia (166). *CSNK1A1* encodes a casein kinase 1α (Ck1α), which is a serine-threonine kinase that fine-tunes the balance between cell proliferation through wingless/integrated WNT/β-catenin signaling and apoptosis mediated by TP53. Hence, if del(5q) MDS cells acquire deletions of TP53, they become resistant to lenalidomide due to complete loss of CK1α and the inability to induce TP53-dependent apoptosis and cell death. Clinicals trials focusing on the combination of lenalidomide and 5-AZA, are in the center of the attention, showing by now favorable results (167, 168).

### 3.3 Signal transduction

A complex interplay of various cytokines has been implied in the regulation of differentiation of the bone marrow in MDS. Growth factors, interleukins, interferons, transforming growth factor beta (TGF-β), and tumor necrosis factor a (TNF-α) are suggested to have stimulatory and/or inhibitory actions on hematopoietic stem cells (169). The physiologic effects of a few of these cytokines are executed by the support of transcription regulators like the janus kinase and signal transducer and activator of transcription (JAK-STAT) pathway and many other pathways (170). Activating mutations of tyrosine kinase or serine/threonine kinase are commonly seen in various myeloid malignancies. Active signaling caused by these mutations, induces cell proliferation and suppresses apoptosis, contributing to the expansion of abnormal clones that have accumulated various mutations.

#### 3.3.1 Signal transduction mutations

Normal cellular growth and differentiation are regulated by the mitogen-activated protein (MAP) and phosphatidylinositol-3 kinase (PI3K)/AKT/mTOR pathways, both of which are activated by the GTPase family proto-oncogene RAS (171, 172). The p38 MAP kinase (MAPK) pathway in HSCs can also be upregulated TGF-β which transduces gene expression signaling *via* phosphorylation of SMAD proteins (173). Mutations in neuroblastoma RAS viral oncogene homolog (*NRAS*) and kirsten rat sarcoma virus gene (*KRAS*) are the most frequent. Both mutations occur at known hotspots, including p.G12, p.G13, and p.Q61. NRAS is found to be mutated in 4-6% of MDS and is shown to predict a phenotype with increased marrow myeloblasts, lower OS, and increased risk of transformation to AML (174), with mutations in KRAS detected in no higher than 1% of cases (175, 176). FLT3 and v-kit Hardy-Zuckerman 4 feline sarcoma viral oncogene homolog (*KIT*) mutations, commonly seen in AML, occur in less than 5% in MDS (177). Neurofibromatosis type 1 *(NF1)*, B-Raf Proto-Oncogene (*BRAF)*, and Protein Tyrosine Phosphatase Non-Receptor Type 11 (*PTPN11)* are rarely mutated. Cbl proto-oncogene (*CBL)* mutations in MDS (5%) are involved in negative modulation and aberrant tyrosine kinase signaling (177).

The V617F JAK2 mutation encoding a cytoplasmic tyrosine kinase, has been described in 5% of MDS associated with megakaryocytic proliferation, and 50% of MDS/MPN overlapping refractory anemia with ring sideroblasts and thrombocytosis (MDS/MPN-RS-T) (37, 63) The overall prognostic significance of *JAK2* is unclear in MDS, but targeting similar to that in MPN is not irrational. Mutations in signal transduction pathways occur in a mutually exclusive manner in individual MDS (24). PTPN11, is part of the RAS family pathway and rarely mutated in adult MDS. The missense mutations include N-SH2 and PTP domains and have been associated with juvenile myelomonocytic leukemia (JMML) and childhood MDS/AML (177, 178). Additionally, it has been found that PTPN11 mutations can be acquired during the progression of MDS or JMML (179).

#### 3.3.2 Targeting signaling transduction pathways

Targeting these mutations has been achieved through direct mutational applications or other involved pathways including PI3K, MEK or MAPK. Both JAK2 and RAS mutations have been targeted with some success in other myeloid diseases, suggesting potential application in MDS as well. Selective, single-agent activity of trametinib, a MEK1/MEK2 inhibitor against RAS-mutated myeloid malignancies, has been evaluated in a phase 2 clinical trial (NCT00920140) and has shown that repeated cycles of the drug were well tolerated, with manageable or reversible toxicities (180). The clinical activity of trametinib is currently being evaluated in a phase 2 clinical trial (NCT04487106) (181), in combination with 5-AZA and venetoclax in patients with AML and HR-MDS. Trials investigating the MAPK pathway include inhibitors of MAPK signaling, namely SCIO-469, ARRY-614, and ezatiostat, with reasonable clinical responses in lower-risk MDS patients (182, 183). TGF-β signaling has been therapeutically targeted by small molecule inhibitor of the TGF-β receptor kinase, LY-2157299 (184), with encouraging preclinical results. Apart from TGF-β receptor kinase inhibition, members of TGF-β superfamily and bone morphogenetic protein ligands have also been targeted by compounds like sotatercept (ACE-011) and ACE-536 (169). The multikinase inhibitor of PI3K, Akt, and PLK1 and RAS mimetic, rigosertib results in decreased tumor cell proliferation. In a phase II dose optimization trial of oral rigosertib in patients with lower-risk MDS (54), different doses of rigosertib were compared in 82 patients, with 15 of 34 eligible patients achieving transfusion independence. In another phase II study (185), rigosertib in combination with 5-AZA led to an overall response rate of 59% in patients who had failed HMA. Another phase III trial (NCT02562443) (186) is ongoing to evaluate the clinical benefit of this subset of patients. Tipifarnib, lonafarnib and a MEK inhibitor (GSK1120212) have shown acceptable toxicity profiles in small studies, and efforts to select mutational subgroups of MDS and AML that may benefit from these inhibitors are underway (186). Although quite common in AML, FLT3 mutations occur rarely in MDS. Despite the approval of midostaurin, sorafenib, gilteritinib, and quizartinib in FLT3-mutated AML this target is less likely to play a role in MDS therapeutics.

### 3.4 Transcription factors

Transcription factors involved in hematopoietic differentiation and HSC regulation are recurrently mutated in MDS. Mutations of the transcriptional factors have been reported to cause impairment of differentiation and maintenance of HSCs of MDS.

#### 3.4.1 Transcription factors mutations

Hematopoietic differentiation involves the activation of lineage-specific gene expression programs by core transcription factors, such as GATA binding protein 2 (GATA2), RUNX1, and CCAAT Enhancer Binding Protein Alpha (CEBPA)f. Recurrent loss-of-function mutations in these molecules occur somatically in MDS and can also be inherited in the germline, thus causing familial bone marrow failure syndromes with a propensity to evolve into myeloid malignancies (28, 29). Familial presentation of MDS due to germline mutations is still unclear but, with the development of NGS techniques, the understanding of pathophysiology of these mutations is in the spotlight. About 45% of MDS/AML families has a mutation in genes including RUNX1, TERT, ANKRD26, CEBPA, DDX41, ETV6, GATA2, TERC, and TP53 (187), with these gene variants having a significant association with disease progression.

The tumor suppressor TP53 plays a key role in response to cellular stress such as DNA damage by activating several protective pathways inducing apoptosis, cell arrest, and DNA repair. It is reported as a unique mutation, being mutually exclusive with other mutations, suggesting its potential role as a driver of mutation in MDS.

*TP53* mutations are seen in 8-13% of MDS, usually (30-50% of cases) in the context of a complex karyotype. They are also associated with chromosomal abnormalities such as deletions of the long arm of chromosome 5 [del(5q)] or chromosome 7 [del(7q)] and high blast percentage, higher-risk disease by IPSS, and adverse prognosis (188). In a retrospective study of patients undergoing pre-transplant genetic profiling, mutations in TP53 were associated with significantly decreased OS (24). *TP53* mutated patients present an unfavorable clinical outcome and a high risk of disease progression (189). In fact, TP53 mutations can divide MDS with complex karyotype into distinct prognostic groups with specific clinical features (60, 190). Another recent study has elucidated the negative prognostic impact of *TP53* mutations in the MDS transplant population, complementary to identifying RUNX1 and ASXL1 as potential markers of poor outcome. *TP53* mutations in association with del(5q) at the early stage of the disease worsens treatment outcomes due to loss of p53 protein function (14). *TP53* mutations seem to have no effect on response to 5-AZA (191). However, a 100% response rate in MDS patients with a TP53 mutation treated with DEC on a 10-day schedule has been demonstrated (192), though this result has not been verified in other cohorts. Moreover, differences in the *TP53* allelic status have been found between low and high-risk patients, since monoallelic *TP53* mutations are commonly seen in patients with lower risk MDS (163). Hence, it is important to investigate the *TP53* allelic status for diagnostic and therapeutic purposes.

#### 3.4.2 Targeting transcription factor mutations

Mutated p53 protein appears to be an important therapeutic target in MDS and AML, therefore restoration of its biological function could be highly beneficial. P53 re-activation and induction of massive apoptosis, {PRIMA-1^Met^ (APR-246 or eprenetapopt)} is a methylated derivative of PRIMA-1, a molecule that reactivates the normal transcription of p53, leading human tumor cells to apoptosis. Mechanistically, APR-246 is a prodrug that binds to cysteine residues of the core domain of mutated p53, resulting in a change of its structure that restores its active formation. Moreover, it induces oxidative stress by depletion of glutathione and inhibition of thioredoxin reductase, thus increasing accumulation of reactive oxygen species and further promoting tumor cell death (193, 194). It has been recently reported that low doses of APR-246 alone or in combination with 5-AZA induce apoptosis in TP53 mutants through p53 reactivation in MDS and AML cell lines (53). In a phase I study including patients with AML, APR-246 monotherapy demonstrated clinical activity with corresponding activation of p53 (51). A recent phase Ib/II study determined the safety and efficacy of APR-246 in combination with 5-AZA in patients with TP53-mutant MDS or low blast count AML (NCT03072043) (52). 5-AZA and APR-246 resulted in a 73% overall response rate with 50% achieving complete remission in the MDS population with a median OS of 10.4 months. These data also support the ongoing phase III multicenter, randomized study of APR-246 in combination with 5-AZA versus 5-AZA alone in patients with *TP53*-mutant MDS (NCT03745716) (195).

Luspatercept (ACE-536) is a novel agent responsible for stimulating the late stage of erythropoiesis through a different mechanism from erythropoietin (196, 197), and is expected to be effective in MDS patients who are refractory to erythropoiesis stimulating agents (ESA). It is a fusion protein of the human immunoglobin G1 (IgG1) which acts through the TGF-β/SMAD pathway, binding to GDF8, GDF11, and activin β, and leading to an increase in erythroid cell maturation by promoting differentiation of erythroblasts. Since TGF-β1 accelerates the terminal maturation of erythroblasts, and luspatercept is supposed to reverse the effects of TGF-β1, it is probably working by preventing the skipping of cell divisions induced by TGF-β1 rather than by supporting maturation. It is approved for the treatment of adult patients with transfusion-dependent anemia due to MDS-RS and an unsatisfactory response to EPO-based therapy, as well as for adult patients suffering from transfusion-dependent anemia associated with beta-thalassemia (198). In lower-risk MDS patients, TGF-β signaling inhibition with luspatercept and sotatercept (ACE-011) has shown activity in clinical trials (194, 199). Interestingly, patients with an SF3B1 mutation seem to respond better to luspatercept than patients without it. A phase 3 trial with 229 randomized transfusion dependent LR-MDS-RS patients, evaluating the erythroid response and transfusion independence of luspatercept (179), showed the effectiveness of the agent for treating ESA-refractory anemia in MDS-RS patients.

### 3.5 The cohesin complex

Cohesin is a multi-subunit protein complex involved in the 3D shaping of the human genome and plays a critical role in chromosome maintenance, transcriptional regulation, and several DNA repair mechanisms (62, 200). The cohesin complex is known to align and stabilize replicated chromosomes prior to cell division.

#### 3.5.1 Cohesin complex mutations

Mutations of the cohesin complex have been reported in various myeloid malignancies, including MDS. Mutations in four components of the cohesin complex are mutually exclusive, indicating that functional disruption of the whole complex is important for the pathogenesis of MDS (201). Recurrent somatic mutations in the cohesin complex are frequent genetic drivers which lead to disturbances in post-replication DNA repair, stabilization of chromosomes, and transcriptional regulation in MDS and other myeloid malignancies (1, 62). Mutations in spliceosome subunits, particularly in PRPF8 and SRF3B1 have also been thought to disrupt sister chromatid cohesion in human cells (202). In overall, it is about loss-of-function nonsense and frameshift mutations, often heterozygous, in a frequency of 12-20% in high risk MDS and secondary AML, mostly indicating poor OS (1, 30, 62). Mutations of the STAG gene have been found in 4-5% of MDS patients, making it the most mutated subunit. STAG2 mutations have been found to be very early events occurring in naïve stem cells and affecting dominant clones (154). Cohesin mutations have been shown to induce abnormal myeloid differentiation and alter the hematopoietic stem cell homeostasis in adult mouse models (203, 204). Therefore, it is assumed that cohesin mutations contribute to the development of MDS through the structural alteration of chromatin and a resulting transcriptional gene deregulation, which is critical for HSCs and their differentiation. A co-occurrence of STAG2 mutation and RAS pathways (NRAS, FLT3) mutations in 15-20% of MDS progressing to AML has also been demonstrated, indicating that the great genomic instability which characterizes the progression from MDS to AML, is associated with these mutations (205).

#### 3.5.2 Targeting the cohesin complex

There are three categories of emerging therapeutic strategies in cohesin mutant cells: direct targeting of cohesin components or its regulators; targeting underlying dysregulated transcription or signaling events; targeting DNA damage repair (DDR) (206, 207). Synthetic lethal interaction between *STAG1* and *STAG2* homologous genes has been identified in several cancer cells (173). Inactivation of *STAG1* leads *STAG2* mutant cells to death. Depletion of the other core subunits RAD21 and structural maintenance of chromosome 3 (SMC3) reduces growth in both wild-type and STAG2 mutant cells as well. In cohesin mutant cancers, targeting DNA methylation or histone modification has an emerging therapeutic potential. It has been shown that myeloid dysplasia patients with *STAG2* or *RAD21* mutations had a significantly better response to DEC and 5-AZA than patients without cohesin mutations (208). Cohesin-deficient cancers cells have shown increased sensitivity to ionizing radiation, because of the cohesin’s role in DNA double-strand break repair. Therefore, using damaging agents to block the underlying DNA repair pathway of cohesin mutant cells has a potential for selective inhibition of these cells. PARP inhibitors (PARPi) benzamide and olaparib have been shown to induce synthetic lethality in cohesin-depleted colon neoplastic cell lines with intrinsic high or low PARP activity (209). Interestingly, a recent study demonstrated increased levels of DNA damage and sensitivity of cohesin-mutant cells to poly(ADP-ribose) polymerase (PARP) inhibition. In the same study, in a mouse model of MDS, *STAG2* mutations arose and were driven by *TET2* mutations and displayed selective depletion of cohesin mutant cells with PARP inhibition *in vivo*. Finally, a shift from STAG2- to STAG1- containing cohesin complexes in cohesin-mutant cells, which was associated with a longer DNA loop and increased interaction with PARP and replication protein A complex was observed (153). Understanding the mechanisms underlying increased sensitivity of cohesin-deficient cells to agents like HMA alone or with combination with other treatment strategies might lead to tailored therapeutics for cohesin mutant cancers.

### 3.6 DNA repair genes

Double-strand breaks (DSB) constitute the most important DNA damage in HSCs. The non-homologous ends-joining (NHEJ) and homologous recombination (HR) repair mechanisms are essentials for ensuring the genomic stability of stem cells. DNA repair is a crucial mechanism for maintaining the integrity of multi-cellular organisms and for ensuring genomic stability (210). Mutations of DNA repair genes may be associated with differences in the efficiency of DNA repair and may influence the risk of developing tumors (55).

#### 3.6.1 DNA repair genes mutations

Ataxia-Telangiesctasia Mutated (ATM) belongs to the phosphatidylinositol 3-kinase-related kinase family. It participates in DNA DSB repair, metabolic regulation, cell cycle checkpoints, and chromatin remodeling (211). It has five major autophosphorylation sites: Ser367, Ser1893, Ser1981, Ser2996, and Thr1885. Once ATM is stimulated by DNA damage, its dimerized form dissociates and converts to monomers, leading to phosphorylation of the above-mentioned residues (212). ATM acts as a cell cycle checkpoint following exposure to stress (213). Somatic mutations have been found in 4% of all samples in a pan-cancer analysis, including missense, loss-of-function and truncating alterations (214).

#### 3.6.2 Targeting DNA damage repair

To study the role of *ATM* in different tumor cell types, recombinase technology techniques have been applied to elucidate how therapies that target ATM may affect cancer compared to normal tissues. Although, at first glance, targeting a tumor suppressor gene could not enhance cancer cell killing, recently a CRISPR screen revealed key FANCONI anemia/BRCA pathway genes as strong synthetic lethality with ATM in the presence of ATM inhibitors (ATMi). Synthetic lethal DNA damage response defects may make the affected tumors more susceptible to ATMi therapy than normal tissues. Growing evidence suggests that *ATM* inactivation may also potentiate the effects of PARPi (215). It has been shown that deficiency of *ATM* in addition to BRCA also confers PARPi sensitivity (216). Moreover, experimental investigations have linked the functional status of p53 and the ability for ATM inactivation to confer radiosensitization (56). It has been previously shown that in HR-MDS and AML, ATM activates NF-κB and that its pharmacological inhibition induces apoptosis in malignant myeloblasts that are freshly isolated from the BM of these patients (217).

### 3.7 Other mutations

SET binding protein 1 gene (*SETBP1*) encodes a protein with 1542 amino-acid residues. SETBP1 is a 170-kDa nuclear protein that binds to SET protein, a protein previously noted to be associated with acute undifferentiated leukemia (218).

#### 3.7.1 SETBP1

The interaction of SETBP1 with the SET nuclear oncoprotein involved in cell division is hypothesized to inhibit various tumor suppressor effects and promote cell proliferation (219). Somatic mutations in *SETBP1* have recently been demonstrated in MDS and mainly affect 858–874 residues. *SETBP1* binds directly to the promoters of *HOXA9* and *HOXA10* resulting in increased expression. Overexpression of *SETBP1* causes inhibition of protein phosphatase 2 (PP2A), leading to cell proliferation. Co-occurrence of *SETBP1* has been noticed with -7/del(7q) and, although it is associated with intermediate prognosis, cooccurrence of SETBP1 with this chromosomal rearrangement might indicate a poor prognostic impact of SETBP1 in MDS (220, 221). In particular, *SETBP1* mutations have been found overrepresented in patients with isolated i (16) (q10) (41-54%) as compared to cases with other cytogenetic rearrangements and were mutually exclusive with *TP53* mutations (222). SETBP1 mutations occur in a frequency of 2-5%, usually in LR-MDS, and are correlated with significantly shorter survival (219). They are somatic gain-of-function mutations and are enriched in patients with ASXL1 mutations, giving a higher risk of transformation to AML (223). It has been previously shown that expression of a constitutively active TGFβ type I receptor (ALK5-TD) inhibits leukemic proliferation of MDS/AML cells with concurrent ASXL1 and SETBP1 mutations.

#### 3.7.2 Targeting SETBP1

The exact mechanisms of SETBP1 mediated leukemogenesis are largely unknown and there are no drugs available to specifically target SETBP1. However, aberrantly reduced acetylation of histone H3 and H4 lysine residues has been found around the promoter regions of multiple TGF-β pathway genes. The HDAC inhibitor vorinostat reverses histone acetylation at these promoter regions and induces transcriptional derepression of the TGF-β pathway genes with a simultaneous growth-inhibition effect in ASXL1 mutants, indicating that HDAC inhibitors will be promising therapeutic drugs for MDS and AML with *ASXL1* and *SETBP1* mutations (224).

### 3.8 B-cell lymphoma 2 (BCL-2)

BCL-2 and its relatives are functionally classified as either antiapoptotic or proapoptotic. Most cells express a variety of antiapoptotic and proapoptotic BCL-2 proteins, and the regulation of their interactions dictates survival or commitment to apoptosis (50).

#### 3.8.1 The role of BCL2 in HR-MDS

The anti-apoptotic protein BCL-2 provides protection against various proapoptotic stresses by blocking cell death induction (225). In HR-MDS harboring *ASXL1, RUNX1, TP53 or EZH2*, clinical remission depends on the quantity of the drug required to overcome the apoptotic resistance. This is due to the finding that the balance between pro-survival and pro-apoptotic proteins of the BCL-2 family is substantially disturbed during disease progression (50). Data from murine models have shown an interaction between *ASXL1, RUNX1, EZH2, TP53* and *BCL-2* expression, suggesting that BCL-2 inhibition might fail to kill progenitors in MDS that harbor any of these mutations (226, 227).

#### 3.8.2 Targeting BCL-2

Since selective BCL-2 inhibition is a promising treatment strategy in hematologic malignancies, the therapeutic impact of venetoclax (formerly known as ABT-199) has been tested in HR-MDS harboring the above mutations. Unlikely to what had been observed in murine models, it has been shown that HR-MDS patient samples specifically undergo cell death in response to venetoclax even when harboring mutations in ASXL1, RUNX1, TP53 or EZH2 (228). The rationale of combining venetoclax with 5-AZA was linked to the existing role of hypomethylating agents in MDS and the promising results observed for the venetoclax/5-AZA combination in older patients with AML (229). The combination of venetoclax and 5-AZA produces a rapid and high rate of bone marrow blast clearance. Data from ongoing phase 3 clinical trials (NCT04401748, NCT04628026) (230, 231) will determine whether combining venetoclax with 5-AZA decreases leukemia stem cell populations, and whether sensitivity in subpopulations correlates with intracellular levels of BCL-2 family proteins. The results of these studies may have a major impact on the way HR-MDS will be managed in the future.

### 3.9 The role of immune checkpoints

Immune checkpoints play an important role in the regulation of immune homeostasis by balancing the stimulatory and inhibitory signals that mediate T-cell immune responses (232). Under normal conditions, immune checkpoints regulate self-tolerance and protect tissues from damage by conducting the immune system response to pathogenic infection.

#### 3.9.1 Immune checkpoints in MDS

Leukemic cells express several checkpoint inhibitor receptors as well as ligands making them potential direct targets for inhibition. Deregulated immune microenvironment has been recognized as a key pathogenic driver of MDS and AML, causing high rate of apoptosis in LR-MDS and immunosuppression in HR-MDS and AML (233). Several cytokines that are abnormally expressed in MDS can induce their expression, which together represent an immune checkpoint involved in regulating T cells in the peripheral tissues (234). Immune checkpoint molecules, including programmed cell death-1 (PD-1) and programmed cell death ligand-1 (PD-L1), play important roles in oncogenesis by maintaining an immunosuppressive tumor microenvironment. Recently, both molecules have been examined in MDS and AML. Abnormal inflammatory signaling, genetic and epigenetic alterations, interactions between cells, and treatment of patients, all have been involved in deregulating PD-1/PD-L1 signaling in these two diseases. Patients with autoimmune diseases seem to develop MDS in a ratio of 1.5-3.5, indicating that immune deregulation could be implicated in the pathogenesis of MDS.

#### 3.9.2 Immune checkpoints inhibitors (ICIs)

Following the successes with ICI1s in solid tumors (235–237) these agents are being evaluated in hematologic malignancies, including AML and MDS (238). Epigenetic modification through PD-1 inhibition is a new approach in HR-MDS. Abnormal upregulation of PD-L1, PD-L2, PD-1, and cytotoxic T-lymphocyte antigen 4 (CTLA4) in CD34+ cells in MDS patients compared to healthy controls has been reported, suggesting that as a potential mechanism triggering HR-MDS (239). Higher percentage of PD-L1 expression is presented on the HR-MDS blasts compared to LR-MDS (240).

Pidilizumab (MDV9300, formerly CT-011) is a humanized monoclonal IgG1 antibody to PD-1. In a phase I study, pidilizumab was relatively safe, with no dose-limiting or treatment-related adverse events (241). Other relevant PD-1 inhibitors include nivolumab and pembrolizumab that are still being evaluated in phase 1b and 2 trials (NCT01953692, NCT02397720) respectively in MDS/AML patients after HMA failure (242, 243). Several clinical trials are trying to evaluate the combination of PD-1/PDL-1 inhibitors with HMAs, showing mixed responses (NCT03094637, NCT02508870, NCT02775903) (244–249)

Ipilimumab is a human IgG1 isotype monoclonal antibody that binds the CTLA-4, a T-cell receptor that downregulates T cells (250). This agent blocks the inhibitory signal, allowing cytotoxic T cell mediated antitumor immune response. Two studies are actively investigating the role of single agent ipilimumab in relapsed/refractory MDS (NCT02530463, NCT01757639). Preliminary results from the phase II study (NCT02530463) that evaluated multiple cohorts (nivolumab alone, ipilimumab alone, nivolumab with ipilimumab) in MDS patients who had failed first-line therapy with an HMA and combination cohorts (5-AZA with nivolumab, 5-AZA with ipilimumab, and 5-AZA with nivolumab and ipilimumab) in previously untreated intermediate 2/high-risk MDS are available (251). The same study included 15 intermediate/high risk MDS patients failing HMA who received single agent nivolumab in a parallel cohort with no responses noted.

#### 3.9.3 CD47 and the “don’t eat me” signal

Cluster of differentiation 47 (CD47) is a cell surface glycoprotein belonging to the immunoglobulin superfamily, binding to various proteins including integrin, thrombospondin-1, and signal regulatory protein α (SIRPα). The interaction of CD47 with SIRPα triggers a “don’t eat me” signal to the macrophages, inhibiting phagocytosis (252). Thus, overexpression of CD47 enables tumor cells to evade immune surveillance *via* the blockade of phagocytic mechanisms. CD47 blockade alone is not sufficient to trigger macrophage anti-tumor activity. Macrophages also need a pro-phagocytic signal. Accumulating data suggest that CD47-SIRPα is a key immune checkpoint in different cancers including hematological malignancies. CD47 monoclonal antibodies (mAbs) not only block CD47 from engaging SIRPα, but also engage the activating Fc gamma receptor (FcγR) on macrophages. Together they deliver a potent phagocytic signal to macrophages (253). Magrolimab is a monoclonal antibody that blocks CD47 to escape immune surveillance and macrophage-mediated clearance (254). Clinical trials have shown that magrolimab, is safe when administered as monotherapy (255). However, the mechanism underlying this observed protection has not been fully defined. Moreover, a synergy between magrolimab and 5-AZA has also been observed. At present, clinical trials designed to examine whether such a synergy can be seen in patients, and to assess the tolerability and clinical activity of the combination (NCT03248479, NCT04313881) (256, 257) show promising results.

## 4 Germline mutations

The molecular recognition of germline predisposition to hematopoietic neoplasms was formalized in the 2016 revision of the WHO classification of myeloid neoplasms and AML. Germline variants in genes such as *CEBPA, DDX41, RUNX1, ETV6* and *GATA2* should be considered in patients when mutations in these genes are seen in tumor sequencing tests, especially when the mutations are biallelic or consistent with their presence in the germline (264). Estimates suggest that 4-10% of children and young adults with MDS or AML and 4% of adults with AML carry inherited mutations affecting cancer susceptibility genes 259.

### 4.1 CEBPA

The CEBPA gene is located on chromosome 19q13.1 and is essential for cellular growth arrest. The inheritance of a germline *CEBPA* mutation predisposes the development of AML with autosomal dominant inheritance. Familial CEBPA AML shares characteristics with somatic *CEBPA* AML (260). It has become clear that familial AML with germline *CEBPA* mutations involves the inheritance of a single copy of mutated *CEBPA* encoding a granulocyte differentiation factor (261) and that more than 10% of AML with biallelic CEBPA mutations carry a N-terminal frameshift *CEBPA* germline mutation with acquisition of a C-terminal somatic mutation as a second event in the development of AML (262).

### 4.2 DDX41

DDX41 is a receptor belonging to the DEAD/H-box helicase family, encoded by a gene comprising 17 exons on chromosome 5 (263). It takes part in pre-mRNA splicing, mRNA export, transcriptional and translational regulation, ribosome biogenesis and RNA decay (264). *DDX41* mutations have been identified as germline mutations in families with multiple cases of late-onset MDS and/or AML. The majority of germline mutations are frameshift mutations suggesting loss of function with DDX41 acting as a tumor suppressor, and there is a common somatic missense mutation found in a majority of germline mutated tumors. Clinically, *DDX41* mutations lead to the development of high-risk MDS (265). Patients with *DDX41* mutations who develop MDS/AML usually present with leukopenia with or without other cytopenias and macrocytosis, in addition to hypocellular bone marrow with prominent erythroid dysplasia, and a normal karyotype, often leading to erythroleukemia (266). The prognosis of these patients is generally poor. In a limited number of patients, cases with DDX41 mutation may respond to lenalidomide (267), while in general *DDX41*-related myeloid malignancies have been reported to present good outcome (268).

### 4.3 RUNX1

*RUNX1* is located on chromosome band 21q22 and is associated with the development of normal hematopoiesis. It is a key regulator of hematopoietic and bone marrow differentiation. In a study of familial platelet disease with propensity to myeloid malignancies (FPD/AML), an autosomal dominant syndrome characterized by platelet abnormalities and susceptibility to MDS/AML that is caused by the genetic mutation of *RUNX1*, the authors identified germline RUNX1 mutations in five families with a history of MDS/AML (269). Germline *RUNX1* mutations include partial and whole gene deletions and frameshift, stop-gain, and missense mutations and are likely to be an initial factor in the development of AML, in addition to other genetic abnormalities. Different families with germline *RUNX1* mutations exhibit varying risks of developing MDS and AML (11-100%). The median age of patients with such mutations at the onset of MDS/AML is 33 years, which is younger than that of sporadic MDS/AML (270, 271).

### 4.4 ETV6

The ETV6 gene is located on chromosome 12. It encodes an ETS family transcription factor with three functional domains necessary for hematopoiesis and the vascular development. Several studies have described germline *ETV6* variations as a susceptibility factor for hematologic malignancies (272–274). Since the first description of germline *ETV6* mutations, 18 families have been reported. The common phenotype is mild to moderate thrombocytopenia with a variable predisposition to acute lymphoblastic leukemia (ALL), AML, and MDS (275).

### 4.5 GATA2

The GATA2 gene, located on chromosome 3, encodes a zinc finger transcription factor that contains two zinc fingers and a nuclear localization signal. This protein is central in the production and maintenance of HSCs in embryonic and adult hematopoietic processes (276). It has been found that GATA2 germline mutations account for 15% of advanced primary MDS cases and 7% of all cases. Germline mutations in *GATA2* can lead to GATA2 deficiency characterized by a complex multi-system disorder that can present with many manifestations including variable cytopenias, bone marrow failure, MDS/AML, and severe immunodeficiency. Penetrance and expressivity within families is often variable. *GATA2* mutational status does not negatively affect the outcome of MDS or HSCT. Moreover, findings have suggested that abnormal clonal hematopoiesis is a common event in symptomatic germline mutated *GATA2* patients with MDS and those with hypocellular marrows without overt morphologic evidence of dysplasia, possibly indicating a pre-MDS stage warranting close monitoring for disease progression (277).

## 5 Conclusions

The genetic and biologic heterogeneity of MDS poses significant challenges in developing new clinical therapeutics. A plethora of molecular pathway disruptions have been used to explain the disease phenotype, but no exclusive genetic drivers can explain all of its aspects. Novel agents targeting the molecular pathways involved in MDS pathogenesis are currently under development while others are already clinically available. In particular, the emerging discovery of small molecules as inhibitors of mutations in genes implicated in cellular processes such as RNA splicing, DNA methylation, chromatin remodeling, signal, and transcription pathways has aroused significant hope in the management of heterogeneous hematological malignancies. Elucidation of expression of mutated genes at the pre-clinical stage and further identification of drug targets will further lead to a better understanding and control of the stages of disease progression. In this context, the genomic profile should be incorporated into a more individualized approach either in first line treatment or after failure/progression. Individualized gene expression and genome wide association studies would further promise to develop personalized therapeutic approaches with pharmaceutical tailoring of targeted drugs.

## Author contributions

C-NK collected and analyzed data and drafted the manuscript. KK collected and analyzed data. N-AV critically reviewed the manuscript. PD designed and directed the project and critically reviewed the manuscript. All authors contributed to the article and approved the submitted version.

## Conflict of Interest

PD reports honoraria from Pfizer, Sandoz, and Roche. N-AV reports investigational grants and personal fees for presentations and advisory roles from Celgene/Genesis Pharma.

The remaining authors declare that the research was conducted in the absence of any commercial or financial relationships that could be construed as a potential conflict of interest.

## Publisher’s note

All claims expressed in this article are solely those of the authors and do not necessarily represent those of their affiliated organizations, or those of the publisher, the editors and the reviewers. Any product that may be evaluated in this article, or claim that may be made by its manufacturer, is not guaranteed or endorsed by the publisher.
