# Supplementary material for: The genetics of myelodysplastic syndromes and the opportunities for tailored treatments
Source: Front Oncol. 2022 Oct 20;12:989483. doi: 10.3389/fonc.2022.989483 (PMC9630842; doi:10.3389/fonc.2022.989483)
Supplement: Supplementary file 1 [file Table_1.docx]

Supplementary table 1. Clinical trials of investigational and already approved targeted therapies for myelodysplastic syndromes.

| **Target** | **Agent** | **Trial Phase** | **Study design** | **Title** | **Status** | **Results** | **Conditions** | **Trial Identifier** | **Reference** |
| --- | --- | --- | --- | --- | --- | --- | --- | --- | --- |
| *SF3B1* | H3B-8800 | I | Monotherapy | A Study of H3B-8800 (RVT-2001) in Participants with Myelodysplastic Syndromes, Acute Myeloid Leukemia, and Chronic Myelomonocytic Leukemia | R | Dose-dependent target engagement, predictable PK profile  Decreased RBC or platelet transfusion requirements observed in 14% of patients | AML, MDS, CMML | NCT02841540 | [1] |
| *SF3B1*  *U2AF1*  *SRSF2* | CA-4948 | I/II | Monotherapy vs combination with 5-AZA or VEN | Dose Escalation/ Expansion Trial of CA-4948 as Monotherapy and in Combination with Azacitidine or Venetoclax in Patients with AML or MDS | R | ΒΜ blast reductions in 10/12 patients with elevated blast counts at baseline  1 CR, 1 CRi and negative MRD, 1 PR, and 2 ΒΜ CRs | AML, MDS | NCT04278768 | [2] |
| *TET2* | Vitamin C | NA | Vs placebo | Epigenetics, Vitamin C, and Abnormal Blood Cell Formation - Vitamin C in Patients with Low-Risk Myeloid Malignancies | R | NA | MDS, CMML1, Cytopenia | NCT03682029 |  |
|  | Vitamin C | Ib/IIa | Monotherapy | A Phase Ib/IIa Study Evaluating the Safety and Tolerability of Vitamin C in Patients with Intermediate or High-Risk Myelodysplastic Syndrome with TET2 Mutations | R | NA | MDS | NCT03433781 |  |
|  | Azacitidine + vitamin C | II | Combination | TET2 Mutations in Myelodysplastic Syndromes and Acute Myeloid Leukemia with Azacitidine + Ascorbic Acid | C | NA | MDS, MPN, AML | NCT03397173 |  |
| IDH1/2 | Ivosidenib (AG-120) and nivolumab | II | Combination | A Study of the IDH1 Inhibitor AG-120 in Combination with the Checkpoint Blockade Inhibitor, Nivolumab, for Patients with IDH1 Mutated Relapsed/Refractory AML and High Risk MDS | W | NA | MDS, AML | NCT04044209 |  |
|  | Olutasidenib (FT-2102) in combination with ASTX727 | I/II | Combination | ASTX727 and FT-2102 in Treating IDH1-Mutated Recurrent/Refractory Myelodysplastic Syndrome or Acute Myeloid Leukemia | W | NA | AML, MDS, R/R-AML, R/R-MDS | NCT04013880 |  |
|  | Ivosidenib (AG-120) with IC vs placebo | III | Vs placebo | A Study of Ivosidenib or Enasidenib in Combination with Induction Therapy and Consolidation Therapy, Followed by Maintenance Therapy in Patients with Newly Diagnosed Acute Myeloid Leukemia or Myedysplastic Syndrome EB2, with an IDH1 or IDH2 Mutation, Respectively, Eligible for Intensive Chemotherapy | R | NA | AML, MDS-EB2 | NCT03839771 |  |
|  | Ivosidenib (AG-120) | II | Monotherapy | IDH1 (AG 120) Inhibitor in Patients with IDH1 Mutated Myelodysplastic Syndrome | R | Well tolerated in MDS patients.  RR, 91%  Particularly effective in treatment naïve HR-MDS with IDH1 mutations | MDS, AML | NCT03503409 | [3] |
|  | VEN with ivosidenib | I/II | With or without 5-AZA | Ivosidenib and Venetoclax with or without Azacitidine in Treating Patients with IDH1 Mutated Hematologic Malignancies | R | NA | AML, MDS/MPN, R/R-AML | NCT03471260 |  |
|  | Ivosidenib (AG-120) in combination with 5-AZA | III | Combination | Study of AG-120 (Ivosidenib) vs. Placebo in Combination with Azacitidine in Patients with Previously Untreated Acute Myeloid Leukemia with an IDH1 Mutation | A, NR | NA | ND-AML, sAML | NCT03173248 |  |
|  | IDH305 | I | Monotherapy | A Dose Finding Study of IDH305 with Standard of Care in IDH1 Mutant Acute Myeloid Leukemia | W | NA | IDH1^mut^ AML | NCT02826642 |  |
|  | Olutasidenib (FT-2102) | I/II | Alone or in combination with azacytidine or cytarabine | Open-label Study of FT-2102 with or without Azacitidine or Cytarabine in Patients with AML or MDS with an IDH1 Mutation | R | FT-2102 has shown favorable safety, PK/PD, and clinical activity.  Single agent CR/CRi, 38% Combination CR/CRi, 27% | IDH1^mut^ AML, MDS | NCT02719574 | [4] |
|  | Ivosidenib (AG-120) and enasidenib (AG-221) | I | Combination with IC | Safety Study of AG-120 or AG-221 in Combination with Induction and Consolidation Therapy in Participants with Newly Diagnosed Acute Myeloid Leukemia (AML) with an IDH1 and/or IDH2 Mutation | A, NR | Ivosidenib, CR 55%  Enasidenib, CR 47%  In patients with CR/CRi/CRp   - under ivosidenib: 39% IDH1 mutation clearance - under enasidenib: 23% IDH2 mutation clearance | IDH1/IDH2^mut^ ND-AML, sAML, MDS (AML) | NCT02632708 | [5] |
|  | AG881 | I | Monotherapy | Study of Orally Administered AG-881 in Patients with Advanced Hematologic Malignancies with an IDH1 and/or IDH2 Mutation | C | NA | IDH1/IDH2^mut^ AML, MDS | NCT02492737 |  |
|  | Ivosidenib (AG-120) | I | Monotherapy | Study of Orally Administered AG-120 in Subjects with Advanced Hematologic Malignancies with an IDH1 Mutation | R | IDH1-mutated R/R AML, ivosidenib at 500 mg QD was associated with   - low frequency of grade ≥3 trAE - transfusion independence - durable remissions - molecular remissions in some patients in CR | IDH1^mut^ R/R-AML, ND-AML, MDS | NCT02074839 | [6] |
|  | LY3410738 | I | Monotherapy | Study of Oral LY3410738 in Patients with Advanced Hematologic Malignancies with IDH1 or IDH2 Mutations | R | NA | IDH1^mut^ AML, MDS, MPN, CMML | NCT04603001 |  |
|  | Enasidenib (AG-221) | II | Monotherapy | IDH2-Post-Allo-Trial for Patients with IDH2-mut Myeloid Neoplasms After Allo-SCT | R | NA | AML, MDS, CMML with IDH2 R172 or R140 | NCT04522895 |  |
|  | Ivosidenib (AG-120) or enasdenib (AG-221) | III | Combination with IC vs placebo | A Study of Ivosidenib or Enasidenib in Combination with Induction Therapy and Consolidation Therapy, Followed by Maintenance Therapy in Patients with Newly Diagnosed Acute Myeloid Leukemia or Myedysplastic Syndrome EB2, with an IDH1 or IDH2 Mutation, respectively, Eligible for Intensive Chemotherapy | R | NA | AML, MDS-EB2 | NCT03839771 |  |
|  | Enasidenib (AG-221) | II | Monotherapy | IDH2 (AG 221) Inhibitor in Patients with IDH2 Mutated Myelodysplastic Syndrome | R | NA | MDS, AML | NCT03744390 |  |
|  | Enasidenib (AG-221) with 5-AZA | II | Combination | Enasidenib and Azacitidine in Treating Patients with Recurrent or Refractory Acute Myeloid Leukemia and IDH2 Gene Mutation | R | NA | AML, CMML, MDS | NCT03683433 |  |
|  | 5-ΑΖΑ with enasidenib (AG-121) | II | Combination | Azacitidine and Enasidenib in Treating Patients with IDH2-Mutant Myelodysplastic Syndrome | A, NR | Enasidenib: well-tolerated with promising efficacy | AML, MDS-EB2 | NCT03383575 | [7] |
|  | AG881 | I | Monotherapy | Study of Orally Administered AG-881 in Patients with Advanced Hematologic Malignancies with an IDH1 and/or IDH2 Mutation | C | NA | AML, MDS | NCT02492737 |  |
|  | Enasidenib (AG-221) | I/II | Monotherapy | Phase 1/2 Study of Enasidenib (AG-221) in Adults with Advanced Hematologic Malignancies with an Isocitrate Dehydrogenase Isoform 2 (IDH2) Mutation | A, NR | Continuous daily enasidenib: well-tolerated  Hematologic responses in AML after failure to previous treatment | Hematologic Neoplasms | NCT01915498 | [8] |
|  | CPX-351 with ivosidenib (AG-120) | II | Combination | CPX-351 and Ivosidenib for the Treatment of IDH1 Mutated Acute Myeloid Leukemia or High-Risk Myelodysplastic Syndrome | R | NA | AML, MDS, MPN | NCT04493164 |  |
|  | Ivosidenib (AG-120) with IC | I | Combination | Ivosidenib and Combination Chemotherapy for the Treatment of IDH1 Mutant Relapsed or Refractory Acute Myeloid Leukemia | NR | NA | R/R-AML, R/R-MDS | NCT04250051 |  |
|  | APR-548 with 5-AZA | I | Combination | APR-548 in Combination with Azacitidine for the Treatment of TP53 Myelodysplastic Syndromes (MDS | R | NA | MDS | NCT04638309 |  |
| P53 | APR-246 with 5-AZA | II | Combination | APR-246 in Combination with Azacitidine for TP53 Mutated AML (Acute Myeloid Leukemia) or MDS (Myelodysplastic Syndromes) Following Allogeneic Stem Cell Transplant | C | NA | AML, MDS | NCT03931291 |  |
|  | APR-246 with 5-AZA | III | Combination | APR-246 & Azacitidine for the Treatment of TP53 Mutant Myelodysplastic Syndromes (MDS) | A, NR | Trial did not meet primary endpoint: CR rates in combination arm superior but not statistically significant. | MDS | NCT03745716 | [9] |
|  | APR-246 with 5-AZA | I/II | Combination | Study of the Safety and Efficacy of APR-246 in Combination with Azacitidine | NA | Combination: safe with potentially higher ORR and CR rate, and longer OS than reported with 5-AZA alone. | MDS, AML, CMML, MPN | NCT03588078 | [10] |
|  | APR-246 with 5-AZA | I/II | Combination | Phase 1b/2 Safety and Efficacy of APR-246 w/Azacitidine for treatment of TP53 Mutant Myeloid Neoplasms | C | Combination: well-tolerated with high rates of clinical response and molecular remissions | MDS, AML, CMML, MPN | NCT03072043 | [11] |
|  | Omacetaxine with VEN | I/II | Combination | Omacetaxine and Venetoclax for the Treatment of Relapsed or Refractory Acute Myeloid Leukemia or Myelodysplastic Syndrome Harboring Mutant RUNX1 | R | NA | R/R AML, R/R MDS | NCT04874194 |  |
| RUNX1 | Pactrinib (SB1518) | II | Monotherapy | SB1518 for Patients with Myelodysplastic Syndrome (MDS) | T | NA | Leukemia | NCT01436084 |  |
| JAK2 | Pactrinib (SB1518) | I/II | Monotherapy | A Phase 1/2 Study of SB1518 for the Treatment of Advanced Myeloid Malignancies | C | Well-tolerated  Lack of substantial myelosuppression, manageable AE  Clinical activity in MF | AML, CMML, MDS, MF | NCT00719836 | [12] |
|  | Decitabine and Cedazuridine with gilteritinib and VEN | I/II | Combination | ASTX727, Venetoclax, and Gilteritinib for the Treatment of Newly Diagnosed, Relapsed or Refractory FLT3-Mutated Acute Myeloid Leukemia or High-Risk Myelodysplastic Syndrome | R | NA | AML, MDS | NCT05010122 |  |
| FLT3 | Azacitidine with quizartinib | I/II | Combination | Azacitidine and Quizartinib for the Treatment of Myelodysplastic Syndrome or Myelodysplastic/Myeloproliferative Neoplasm with FLT3 or CBL Mutations | R | NA | MDS, MDS/MPN | NCT04493138 |  |
|  | Gilteritinib | I/II | alone or in combination with 5-AZA and VEN | Azacitidine, Venetoclax, and Gilteritinib in Treating Patients with Recurrent/Refractory FLT3-Mutated Acute Myeloid Leukemia, Chronic Myelomonocytic Leukemia, or High-Risk Myelodysplastic Syndrome/Myeloproliferative Neoplasm | R | NA | R/R AML, R/R CMML, R/R MDS/MPN | NCT04140487 |  |
|  | Selinexor with sorafenib | I/II | Combination | Phase I/II, Study of Selective Inhibitor of Nuclear Export (SINE) Selinexor (KPT-330) + Sorafenib in Acute Myeloid Leukemia | C | Combination induced CR/PR in 6/14 patients with R-AML, who had received a median of three prior therapies | AML | NCT02530476 | [13] |
|  | Decitabine with midostaurin | II | Combination | Decitabine and Midostaurin in Treating Older Patients with Newly Diagnosed Acute Myeloid Leukemia | T | NA | AML | NCT01846624 |  |
|  | Pactrinib (SB1518) | II | Monotherapy | SB1518 for Patients with Myelodysplastic Syndrome (MDS) | T | NA | MDS | NCT01436084 |  |
|  | Bortezomib with midostaurin and IC | I | Combination | Phase I Combination of Midostaurin, Bortezomib, and Chemo in Relapsed/Refractory Acute Myeloid Leukemia | C | Combination: active in RR-AML and associated with expected drug-related toxicities | R/R AML | NCT01174888 | [14] |
|  | RAD001 with PKC412 | I | Combination | RAD001 in Combination with PKC412 in Patients with Relapsed, Refractory or Poor Prognosis AML or MDS | A, NR | NA | AML, MDS | NCT00819546 |  |
|  | Gilteritinib | III | Gilteritinib vs  Midostaurin in combination With IC | A Study of Gilteritinib Versus Midostaurin in Combination with Induction and Consolidation Therapy Followed by One-year Maintenance in Patients with Newly Diagnosed Acute Myeloid Leukemia or Myelodysplastic Syndromes with Excess Blasts-2 with FLT3 Mutations Eligible for Intensive Chemotherapy | W | NA | AML, MDS-EB2 | NCT04027309 |  |
|  | E6201 | I/II | Monotherapy | A Study of E6201 for the Treatment of Advanced Hematologic Malignancies with FLT3 and/or Ras Mutations | T | NA | AML, MDS, CMML | NCT02418000 |  |
|  | Sorafenib with 5-AZA | II | Combination | Sorafenib Plus 5-Azacitidine Initial Therapy of Patients with Acute Myeloid Leukemia (AML) and High-Risk Myelodysplastic Syndrome (MS) with FLT3-ITD Mutation | C | Combination well tolerated and effective in older untreated *FLT3*-ITD mutated AML. | AML, HR-MDS | NCT02196857 | [15] |
|  | PKC412I/traconazole | I/II (IIb) | Combination | PKC412 in Participants with Acute Myeloid Leukemia or with Myelodysplastic Syndrome (CPKC412A2104 Core); and PKC412 in Participants with Acute Myeloid Leukemia or with Myelodysplastic Syndrome with Either Wild Type or Mutated FMS-like Tyrosine Kinase 3 (FLT3) (CPKC412A2104E1 and CPKC412A2104E2) | C | NA | AML, MDS | NCT00045942 |  |
|  | 5-AZA, nivolumab, DEC, midostaurin | II/III | 5-AZA with or without nivolumab or midostaurin, or decitabine and cytarabine Alone | Azacitidine with or without Nivolumab or Midostaurin, or Decitabine and Cytarabine Alone in Treating Older Patients with Newly Diagnosed Acute Myeloid Leukemia or High-Risk Myelodysplastic Syndrome | A, NR | NA | AML, HR-MDS | NCT03092674 |  |
|  | Midostaurin with 5-AZA | I/II (II) | Combination | Midostaurin and Azacitidine in Treating Elderly Patients with Acute Myelogenous Leukemia | C | The addition of midostaurin to standard chemotherapy prolonged OS (22% lower risk of death) and EFS in patients with AML and FLT3 mutation. | ND AML | NCT01093573 | [16] |
|  | Sorafenib with cytarabine | I/II | Combination | Sorafenib and Low Dose Cytarabine in Older Patients with Acute Myeloid Leukemia or High-Risk Myelodysplastic Syndrome | C | NA | AML, HR-MDS | NCT00516828 |  |
|  | VEN with 5-AZA | III | VEN with 5-AZA vs placebo | Study of Venetoclax Tablet with Intravenous or Subcutaneous Azacitidine to Assess Change in Disease Activity In Adult Participants with Newly Diagnosed Higher-Risk Myelodysplastic Syndrome (Verona) | R | NA | MDS | NCT04401748 | [17] |
| BCL-2 | VEN | III | VEN with IC vs placebo with IC | Phase III Study of Induction and Consolidation Chemotherapy with Venetoclax in Patients with Newly Diagnosed AML or MDS-EB-2 | NR | NA | AML, MDS | NCT04628026 | [18] |
|  | Pembrolizumab with lenalidomide | I | Pembrolizumab with or without lenalidomide | A Trial of Pembrolizumab (MK-3475) in Participants with Blood Cancers (MK-3475-013/KEYNOTE-013) | C | Pembrolizumab: manageable safety and clinical activity in patients with HMA-refractory MDS.  No patient achieved CR or PR  Median OS: 6.0 months | MDS, MM, HL, NHL, DLBCL, FL, PMBCL | NCT01953692 | [19]. |
| ICs | Nivolumab, 5-ΑΖΑ, ipilimumab | II | Nivolumab with 5-AZA with or without ipilimumab | Nivolumab and Azacitidine with or without Ipilimumab in Treating Patients with Refractory/Relapsed or Newly Diagnosed Acute Myeloid Leukemia | R |  | R/R AML | NCT02397720 |  |
|  | 5-AZA with pembrolizumab | II | Combination | Azacitidine and Pembrolizumab in Treating Patients with Myelodysplastic Syndrome | A, NR | Combination: relatively safe and well-tolerated. Combination: may have antitumor activity in patients who failed HMA, but no significant improvement in OS was observed. | MDS | NCT03094637 | [20] |
|  | Atezolizumab, 5-AZA | I | Atezolizumab alone or in combination with 5-AZA | A Study of Atezolizumab Administered Alone or in Combination with Azacitidine in Participants with Myelodysplastic Syndromes | C | High death rate and poor efficacy do not support a favorable risk-benefit profile for atezolizumab as a single agent or in combination with 5-AZA in R/R or HMA-naïve MDS. | MDS | NCT02508870 | [21] |
|  | Azacitidine with durvalumab | II | Combination | An Efficacy and Safety Study of Azacitidine Subcutaneous in Combination with Durvalumab (MEDI4736) in Previously Untreated Adults with Higher-Risk Myelodysplastic Syndromes (MDS) or in Elderly Patients with Acute Myeloid Leukemia (AML) | A, NR | DNA methylation, mutational status, and PD-L1 expression not associated with response  Combination in older patients: feasible but no improvement in clinical efficacy compared with 5-AZA monotherapy | AML, MDS | NCT02775903 | [22] |
|  | Nivolumab, ipilimumab, 5-AZA | II | Nivolumab and/or ipilimumab with or without 5-AZA | Nivolumab and/or Ipilimumab with or without Azacitidine in Treating Patients with Myelodysplastic Syndrome | R | NA | MDS | NCT02530463 |  |
|  | Ipilimumab | I | Monotherapy | Ipilimumab in Treating Patients with Relapsed or Refractory High-Risk Myelodysplastic Syndrome or Acute Myeloid Leukemia | C | Ipilimumab dosed at 3 mg/kg in patients with MDS after HMA failure is safe but has limited efficacy as a monotherapy. | R/R HR-MDS, R/R AML | NCT01757639 | [23] |
| Abbreviations: A, active; 5-AZA, azacytidine; Allo-SCT, allogenic stem cell transplantation; AML, acute myeloid leukemia; BCL-2, B cell lymphoma 2; BM, bone marrow; C, completed; CMML, chronic myelomonocytic leukemia; CR, complete remission; CRi, complete remission with incomplete hematologic recovery; CTLA-4, cytotoxic T-lymphocyte associated protein 4; DLBCL, diffuse large B cell lymphoma; EFS, event-free survival; ENA, enasidenib; FL, follicular lymphoma; FLT3, fms related receptor tyrosine kinase 3; HL, Hodgkin lymphoma; HMA, hypomethylating agent; HR, high risk; IC, intensive chemotherapy; ICs, immune checkpoints; IDH1/2, isocitrate dehydrogenase ½; IDH1mut, IDH1 mutant; IDH2mut, IDH2 mutant; IVO, ivosidenib; JAK2, janus kinase 2; MDS, myelodysplastic syndrome; MDS-EB2, MDS-excess blasts 2; MEC, minimum effective concentration; MF, myelofibrosis; MM, multiple myeloma; MPN, myeloproliferative neoplasm; MRD, minimal residual disease; NA, not applicable; ND, newly diagnosed; NHL, non-Hodgkin lymphoma; NR, not recruiting; ORR, overall response rate; OS, overall survival; p53, protein 53; PD, pharmacodynamics; PK, pharmacokinetics; PMBCL, primary mediastinal large B-cell lymphoma; PR, partial remission; QD, once daily; R, recruiting; RBC, red blood cells; RR, response rate; R/R, relapsed/refractory; RUNX1, runt-related transcription factor 1; sAML, secondary AML; SF3B1, splicing factor 3b subunit 1; SRSF2, serine and arginine rich splicing factor 2; TET2, tet methylcytosine dioxygenase 2; TP53, tumor protein 53; trAE, treatment-related adverse event; U2AF1, U2 Small Nuclear RNA Auxiliary Factor 1; VEN, venetoclax; W, withdrawn | | | | | | | | | |

References

1. Steensma D, Wermke M, Klimek V, Greenberg P, Font P, Komrokji R et al., Results of a Clinical Trial of H3B-8800, a Splicing Modulator, in Patients with Myelodysplastic Syndromes (MDS), Acute Myeloid Leukemia (AML) or Chronic Myelomonocytic Leukemia (CMML). *Blood* 2019; 134 (Supplement_1): 673. doi: <https://doi.org/10.1182/blood-2019-123854>
2. Curis announces positive updated data from ongoing phase 1/2 study of CA-4948 monotherapy in patients with relapsed or refractory acute myeloid leukemia and myelodysplastic syndromes. News release. Curis, Inc.; June 11, 2021. Accessed January 7, 2022
3. Sebert M, Cluzeau Th, Rauzy OB, Stamatoulas Bastard A, Dimicoli-Salazar S, Thepot S et al., Ivosidenib Monotherapy Is Effective in Patients with IDH1 Mutated Myelodysplastic Syndrome (MDS): The Idiome Phase 2 Study By the GFM Group. *Blood* 2021; 138 (Supplement 1): 62. doi: <https://doi.org/10.1182/blood-2021-146932>
4. Watts J, Baer M, Lee S, Yang J, Dinner SN, Prebet TH et al., A phase 1 dose escalation study of the IDH1m inhibitor, FT-2102, in patients with acute myeloid leukemia (AML) or myelodysplastic syndrome (MDS). Journal of Clinical Oncology 2018 36:15_supplement, 7009-7009
5. Stein EM, DiNardo CD, Fathi AT, Mims AS, Pratz KW, Savona MR et al., Ivosidenib or enasidenib combined with intensive chemotherapy in patients with newly diagnosed AML: a phase 1 study. Blood. 2021;137(13):1792-1803. doi: 10.1182/blood.2020007233.
6. DiNardo CD, Stein EM, de Botton S, Roboz GJ, Altman JK, Mims AS et al., Durable Remissions with Ivosidenib in IDH1-Mutated Relapsed or Refractory AML. N Engl J Med. 2018;378(25):2386-2398. doi: 10.1056/NEJMoa1716984.
7. Venugopal S, Dinardo CD, Takahashi K, Konopleva M, Loghavi S, Borthakur G, et al., Phase II study of the IDH2-inhibitor enasidenib in patients with high-risk IDH2-mutated myelodysplastic syndromes (MDS), Journal of Clinical oncology 2021 39, no. 15_supplement_7010-7010
8. Stein EM, DiNardo CD, Pollyea DA, Fathi AT, Roboz GJ, Altman JK et al., Enasidenib in mutant *IDH2* relapsed or refractory acute myeloid leukemia. Blood. 2017;130(6):722-731.
9. Aprea Therapeutics announces results of primary endpoint from phase 3 trial of eprenetapopt in TP53 mutant myelodysplastic syndromes (MDS). <https://ir.aprea.com/news-releases/news-release-details/aprea-therapeutics-announces-results-primary-endpoint-phase-3> 2020
10. Cluzeau T, Sebert M, Rahmé R, Cuzzubbo S, Lehmann-Che J, Madelaine I,et al. Eprenetapopt plus azacitidine in TP53-mutated myelodysplastic syndromes and acute myeloid leukemia: A phase II study by the Groupe Francophone des Myélodysplasies (GFM). *J Clin Oncol*. 2021. Online ahead of print.
11. Sallman DA, DeZern AE, Garcia-Manero G, Steensma DP, Roboz GJ, Sekeres MA et al., Eprenetapopt (APR-246) and Azacitidine in *TP53*-Mutant Myelodysplastic Syndromes. J Clin Oncol. 2021;39(14):1584-1594. doi: 10.1200/JCO.20.02341.
12. Verstovsek S, Odenike O, Singer JW, Granston T, Al-Fayoumi S, Deeg HJ. Phase 1/2 study of pacritinib, a next generation JAK2/FLT3 inhibitor, in myelofibrosis or other myeloid malignancies. J Hematol Oncol. 2016;9(1):137
13. Zhang W, Ly C, Ishizawa J, Mu H, Ruvolo V, Shacham S, et al., Combinatorial targeting of XPO1 and FLT3 exerts synergistic anti-leukemia effects through induction of differentiation and apoptosis in FLT3-mutated acute myeloid leukemias: from concept to clinical trial. Haematologica. 2018 103(10):1642-1653. doi: 10.3324/haematol.2017.185082.
14. Walker AR, Wang H, Walsh K, Bhatnagar B, Vasu S, Garzon R et al., Midostaurin, bortezomib and MEC in relapsed/refractory acute myeloid leukemia. Leuk Lymphoma. 2016;57(9):2100-8. doi: 10.3109/10428194.2015.1135435.
15. Ohanian M, Garcia-Manero G, Levis M, Jabbour E, Daver N, Borthakur G et al., Sorafenib plus 5-azacytidine (AZA) in older untreated *FLT3*-ITD mutated AML. Acute Myeloid Leukemia. Am J Hematol. 2018;93(9):1136-1141. doi: 10.1002/ajh.25198.
16. Stone RM, Mandrekar SJ, Sanford BL, Laumann K, Geyer S, Bloomfield CD et al., Midostaurin plus Chemotherapy for Acute Myeloid Leukemia with a FLT3 Mutation. N Engl J Med. 2017;377(5):454-464
17. Zeidan AM, Suen Garcia J, Fenaux P, Platzbecker U, Miyazaki Y, Xiao ZJ et al., 3 VERONA study of venetoclax with azacitidine to assess change in complete remission and overall survival in treatment-naïve higher-risk myelodysplastic syndromes. Journal of Clinical Oncology 2021 39:15_supplement_TPS7054-TPS7054
18. Juliusson G. Venetoclax with intensive chemotherapy in younger patients with acute myeloid leukaemia. Lancet Haematol. 2022;(5):e317-e318. doi: 10.1016/S2352-3026(22)00100-4.
19. Garcia-Manero G, Ribrag V, Zhang Y, Farooqui M, Marinello P, Smith BD. Pembrolizumab for myelodysplastic syndromes after failure of hypomethylating agents in the phase 1b KEYNOTE-013 study. Leuk Lymphoma. 2022;1-9. doi: 10.1080/10428194.2022.2034155.
20. Chien K, Borthakur G, Naqvi K, Daver N, Montalban Bravo G,Cortes J et al., Final Results from a Phase II Study Combining Azacitidine and Pembrolizumab in Patients with Higher-Risk Myelodysplastic Syndrome after Failure of Hypomethylating Agent Therapy. Blood 2020; 136 (Supplement 1): 23–24. doi: https://doi.org/10.1182/blood-2020-141100
21. Gerds AT, Scott BL, Greenberg P, Lin TL, Pollyea DA, Verma A et al., Atezolizumab alone or in combination did not demonstrate a favorable risk-benefit profile in myelodysplastic syndrome. Blood Adv. 2022;6(4):1152-1161
22. Zeidan AM, Boss I, Beach CL, Copeland WB, Thompson E, Fox BA et al., A randomized phase 2 trial of azacitidine with or without durvalumab as first-line therapy for older patients with AML. Blood Adv. 2022;6(7):2219-2229
23. Zeidan AM, Knaus HA, Robinson TM, Towlerton AMH, Warren EH, Zeidner JF et al., A Multi-center Phase I Trial of Ipilimumab in Patients with Myelodysplastic Syndromes following Hypomethylating Agent Failure. Clin Cancer Res. 2018;24(15):3519-3527. doi: 10.1158/1078-0432.CCR-17-3763.
